# Magnetostratigraphic constraints on the late Ediacaran paleomagnetic enigma

**DOI:** 10.1126/sciadv.ady3258

**Published:** 2025-10-03

**Authors:** James S. Pierce, David A. D. Evans, Dana E. Polomski, Nasrrddine Youbi, Mohamed A. Mediany, Jihane Ounar, Rachid Oukhro, M. Ahmed Boumehdi, Justin V. Strauss, C. Brenhin Keller, Andres Gärtner, Maria Ovtcharova, Jörn-Frederik Wotzlaw, Ulf Linnemann

**Affiliations:** ^1^ Yale University, 210 Whitney Avenue, New Haven, CT 06511, USA.; ^2^ Department of Geology, Faculty of Sciences Semlalia, Cadi Ayyad University, Prince Moulay Abdellah Boulevard, Marrakech, Morocco.; ^3^ Instituto Dom Luiz, Faculdade de Ciências, Universidade de Lisboa, 1749-016 Lisboa, Portugal.; ^4^ Faculty of Geology and Geography, Tomsk State University, 36 Lenin Ave, Tomsk 634050, Russia.; ^5^ Department of Earth Sciences, Dartmouth College, Hanover, NH 03755, USA.; ^6^ Senckenberg Naturhistorische Sammlungen Dresden, Museum für Mineralogie und Geologie, Sektion Mineralogie/Isotope Forensics, Königsbrücker Landstraße 159, D-01159 Dresden, Germany.; ^7^ Department of Earth Sciences, University of Geneva, 1211 Geneva, Switzerland.; ^8^ Institute of Geochemistry and Petrology, ETH Zurich, 8092 Zurich, Switzerland.; ^9^ Senckenberg Naturhistorische Sammlungen Dresden, GeoPlasmaLab, Königsbrücker Landstraße 159, 01109 Dresden, Germany.

## Abstract

Paleogeography of the Ediacaran Period has remained poorly understood because of paleomagnetic studies commonly yielding perplexing or conflicting data. Here, we report new magnetostratigraphic data from the Ediacaran Ouarzazate Group in the Anti-Atlas Mountains of Morocco, which have primary magnetizations supported by a positive conglomerate test and stratigraphically consistent directions within volcanic units across multiple localities. Comprehensive magnetostratigraphic sampling shows highly variable directions, consistent with a rapidly changing geomagnetic field along a longitudinally preferred band. High-precision geochronology constrains the geomagnetic variability to ~568 to 562 million years and suggests rates that are likely too rapid for true polar wander or plate tectonic interpretations. Comparison of igneous- and sedimentary-derived data, using a new statistical approach combining Bingham and Fisher distributions, indicates a high-inclination paleomagnetic direction that is compatible with independent evidence for regional glaciation. Our analysis produces a late Ediacaran paleogeographic reconstruction that is consistent with paleomagnetic and geologic constraints.

## Teaser

A paleomagnetic analysis reveals rapid changes in the Ediacaran geomagnetic field and produces a robust paleogeography.

## INTRODUCTION

Paleomagnetic studies of Ediacaran rocks from several cratons have consistently yielded discordant data, suggesting rapid changes in paleomagnetic direction over short geologic time spans (*1*). Studies from Laurentia, Baltica, Australia, and the West African Craton show anomalously fast apparent polar wander and challenge the validity of the geocentric axial dipole (GAD) hypothesis, a standard assumption for plate reconstructions in deep time (*2*–*5*). In Laurentia, for example, many igneous-derived paleomagnetic datasets contain large directional variability that is commonly split into subsets (*4*, *6*, *7*) with ambiguous paleogeographic implications (*8*). Compounding this paleogeographic ambiguity, magnetostratigraphic studies have shown that the late Ediacaran magnetic field was rapidly reversing (*9*, *10*) and paleointensity data suggest an extremely weak field (*11*).

Several hypotheses have been proposed to explain these paleomagnetic results, including very fast plate tectonics, true polar wander (TPW) (*12*), unrecognized overprints (*13*), and/or an irregular magnetic field (*2*, *3*, *5*). However, none fully satisfy the available data (*1*). For example, plate tectonics cannot reasonably account for rates of apparent polar wander far exceeding ~20 cm/year. TPW, where the solid outer part of the Earth rotates relative to a fixed spin axis, may permit faster speeds than plate tectonics and has frequently been cited to explain these data; however, several Ediacaran studies, including this study, show apparent polar wander rates in the hundreds of centimeters per year, far exceeding theoretical estimates of 66 cm/year suggested for maximum TPW velocity (*14*, *15*). Unrecognized overprints have been proposed for some discordant Ediacaran datasets (*13*), but these are unlikely to explain all of the data discordances globally. A nondipolar or chaotic magnetic field has been proposed, perhaps including a nonnegligible equatorial dipole component or the recording of enhanced paleosecular variation (PSV) (*2*, *3*, *5*), but such models lack a robust spatiotemporal framework of geomagnetic field variability.

The Ediacaran Ouarzazate Group in the Anti-Atlas Mountains of Morocco is an ~2.5-km-thick succession of volcanic and subordinate sedimentary rocks formed at the northwestern (present coordinates) margin of the West African Craton between 580 and 540 million years (Ma). The magmatism has been attributed to continental arc volcanism and eventual slab break-off with its subsequent asthenospheric upwelling, in addition to possible influence from passage over a mantle plume (*16*–*19*). The Ouarzazate Group of the Bou Azzer inlier, which was examined herein, contains decameter-scale silicic to intermediate volcanic flows and tuffs with subordinate siliciclastic rocks, interpreted as an outflow ignimbrite succession flanking an adjacent unnamed caldera located north of the Anti-Atlas Major Fault. Precambrian rocks in the Anti-Atlas Mountains were gently to moderately deformed during the late Paleozoic Hercynian Orogeny, but in the Bou Azzer inlier, they are exquisitely preserved with nearly horizontal structural attitudes and primary mineralogy (*16*, *17*, *19*). A previous paleomagnetic study of similar units in the Ouarzazate Group of this region found primary magnetization carried by magnetite and hematite (*14*). That study generated two reliable paleomagnetic poles that differ significantly and suggested rapid apparent polar wander; however, no single locality contained both directions, leaving questions about their stratigraphic and temporal relationship, as well as the implied rates of geomagnetic variability. Our study uses magnetostratigraphy and high-precision geochronology to determine the validity of the various hypotheses proposed to explain Ediacaran paleomagnetic data.

## RESULTS

**Fig. 1.**
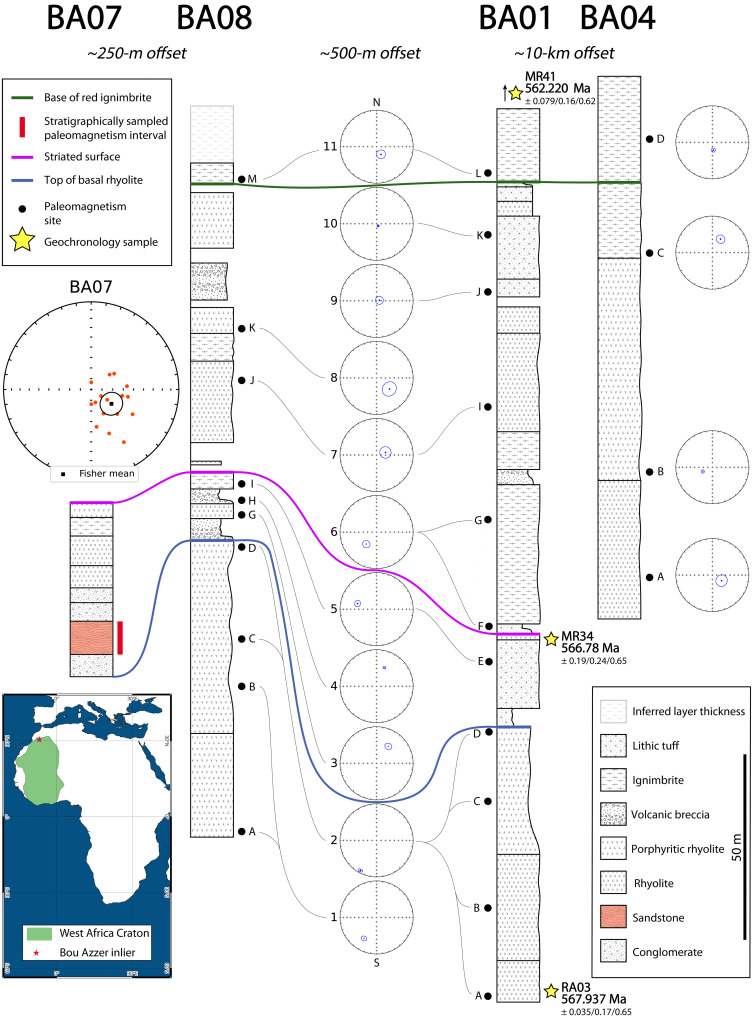
Magneto- and chronostratigraphy of the Ouarzazate Group in the central Bou Azzer inlier, Morocco. The map on the bottom left shows the location of the sampling site on the West African Craton. Stratigraphic sections BA01, BA04, BA07, and BA08 were correlated using distinct marker beds, indicated by the pink, blue, and green lines connecting the measured sections. Paleomagnetic directions from the composite section of BA01 and BA08 are plotted on equal area projections in the center, linked by lines to their stratigraphic position. Paleomagnetic directions from sections BA07 and BA04 are plotted on the far left and far right, respectively. The stratigraphic positions of the geochronology samples presented in this study are indicated by yellow stars and sample names. All directions are corrected for bedding. Here, BA07 directions are not corrected for compaction-induced inclination shallowing.

We identified four stratigraphic sections within the Bou Azzer inlier (BA01, BA04, BA07, and BA08) that contain regional-scale marker horizons, allowing unambiguous correlation (Fig. 1). The bases of sections BA01 and BA08 are ~500 m apart laterally and are easily correlatable lithostratigraphically. Toward the middle of sections BA01 and BA08, striated and grooved surfaces atop a prominent rhyolite unit have been assigned to the late Ediacaran Bou Azzer glaciation (*20*, *21*). Syndepositional faults produced minor stratigraphic variability, including rare preservation of hematite-bearing clastic sedimentary rocks (“red beds”) in a thin succession that we call BA07. Other important marker horizons include a distinctive basal rhyolite flow that we use to correlate the lower parts of the sections and a widespread ignimbrite that preserves fiamme welding textures and caps the sections that we use to correlate across the entire study area. The BA07 red beds lie stratigraphically between the basal rhyolite and the striated and grooved marker horizon. Another section, BA04, is situated ~10 km to the west, providing broader spatial coverage to the dataset.

More than 250 oriented paleomagnetic samples were collected from successions of predominantly volcanic and subordinate sedimentary rocks in the Bou Azzer inlier. Most samples yielded variable present-field or Hercynian partial overprinting on high unblocking-temperature characteristic remanent magnetization (ChRM) vectors (data S1 and S2 and fig. S3). Sites were grouped by volcanic unit, stratigraphy, and magnetic directions. Fisher directional means were then calculated for each unit (Fig. 1). ChRM directions shift up-section stratigraphically from shallow to steep and show transitional directions in both polarities that are correlatable to individual stratigraphic units. When plotted together, the site means fall along a great circle trending from southwest to northeast. A conglomerate test on rhyolitic clasts in a terminal Ediacaran unit at the top of the composite section demonstrates that the underlying volcanic rocks have not been remagnetized (fig. S5). Siliciclastic rocks from section BA07 hold stable magnetizations with unblocking temperatures in the range of hematite (>670°C). Their ChRM directions are east to southeast and steep down, similar to directions from the upper part of sections BA01 and BA08.

**Fig. 2.**
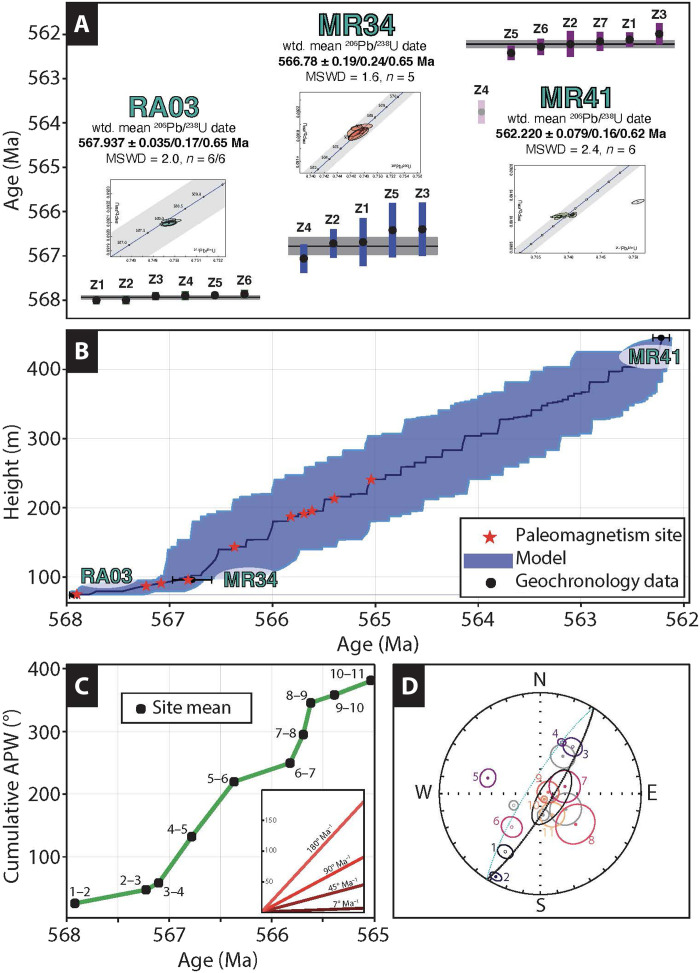
Uranium-lead ages, age model, apparent polar wander rates, and paleomagnetic site means with an overall Bingham mean. (**A**) Uranium-lead results from Ouarzazate Group samples within the measured sections. Sample RA03 is from the basal rhyolite of sections BA01, BA07, and BA08. Sample MR34 is from directly below the grooved surface used for regional correlation. Sample MR41 is from a rhyolite stratigraphically above sections BA01, BA08, and BA07 and directly below a sub-Cambrian conglomerate, which yielded a positive paleomagnetic conglomerate test. (**B**) Bayesian age model for the composite section BA01 extending upward to sample MR41, ~200 m stratigraphically above last paleomagnetism sample. The model incorporates the discrete eruptions of volcanic rocks, shown here by a stepwise age progression moving up stratigraphy. Location of geochronology samples and paleomagnetism samples are denoted by the black circles and red stars, respectively. (**C**) Cumulative apparent polar wander derived from the magnetostratigraphic virtual geomagnetic poles is plotted against site age interpolated using the age model from (B). For reference, the inset illustrates how the slope of apparent polar wander rates of 180°, 90°, 45°, and 7° per Ma would plot. A slope of 7° per Ma was chosen to represent the rate of a fast TPW event [Rose and Buffett, 2017; (*15*)]. (**D**) Site means from composite section BA01/BA08 are plotted on an equal area projection. The site letter names correspond with the equal area plots from Fig. 1. The gray means are from section BA04, which cannot be placed precisely instratigraphic context with BA0, BA07, and BA08. A Bingham mean and ellipse is superimposed along with a great circle that was calculated using the Bingham parameters.

Volcanic units in our measured profiles were sampled for zircon U-Pb geochronology via chemical abrasion–thermal ionization mass spectrometry (CA-TIMS; Materials and Methods). Zircon dates were successfully obtained from three units (Fig. 2): Sample RA03 from the very base of the composite section yields a date of 567.935 ± 0.035/0.17/0.65 Ma, sample MR34 from immediately below the striated and grooved surface purported to mark the Bou Azzer glaciation yields a date of 566.78 ± 0.19/0.24/0.65 Ma, and sample MR41 from directly below the aforementioned terminal Ediacaran conglomerate yields a date 562.220 ± 0.079/0.16/0.62 Ma. Pronounced shifts in paleomagnetic inclinations occur between 567.935 Ma (RA03) and 566.78 Ma (MR34) and continue further up-section, which, if interpreted in a traditional sense of paleolatitude shifts, would correspond to rates on the order of ~100° per Ma, far too rapid for either plate tectonics or TPW (*1*). Because the time constant for liquid motions in the Earth’s outer core is of the order of 10^3^ years, the pronounced paleomagnetic variability in Ouarzazate volcanic strata is most consistent with PSV of the Ediacaran geodynamo. A similar conclusion was made by Robert *et al.* (*5*) from a regional study of the Ouarzazate Group, but our study reports such rapid variability within continuously exposed strata and a precisely ordered temporal sequence (fig. S6).

**Fig. 3.**
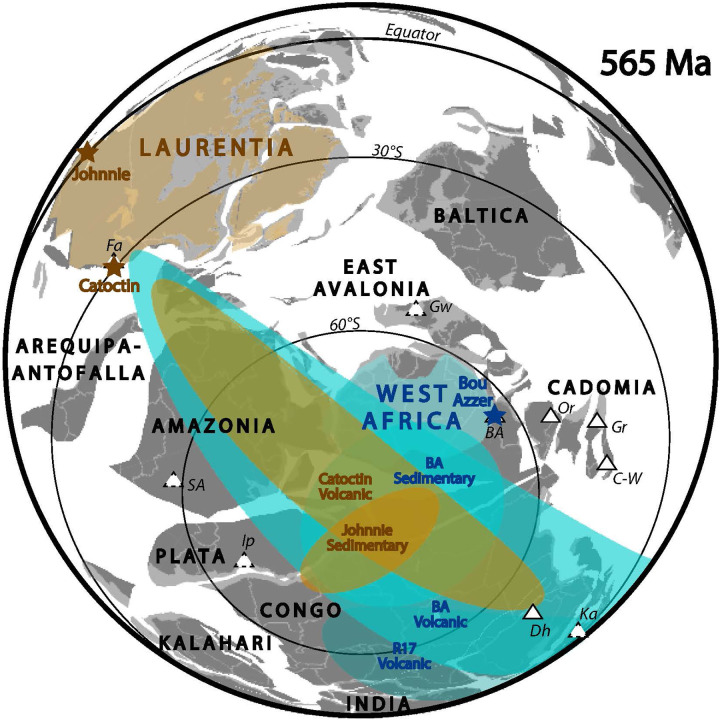
Plate reconstruction of West African Craton (blue) and Laurentia (orange) according to Kent statistical treatment of sedimentary data and Bingham statistical treatment of coeval volcanic data. Sedimentary data provide absolute paleolatitudes, while volcanic data provide relative paleolongitudes assuming a dipolar geometry of enhanced PSV. Stars indicate the paleomagnetic sampling regions. Glaciogenic deposits are noted by triangles with solid/dashed outlines according to high/moderate confidence in both the evidence for ice influence and the assignment to an age of ~565 Ma (*27*). Abbreviations: BA, Bou Azzer (*21*, *22*); C-W, Clanzschwitz-Weesenstein (*26*); Dh, Dhaiqa; Fa, Fauquier; Gr, Granville (*27*); Gw, Gwna; Ip, Iporanga; Ka, Kahar (*64*); Or, Orellana (*26*); SA, Serra Azul (*65*, *66*); R17, Robert *et al.* 2017 (*15*). Positions of additional cratons that lack coeval igneous and sedimentary paleomagnetic data from this time interval, with modern coastlines and political borders shown for clarity, are given according to a reconstruction by D. Evans and B. Eglington based on global tectonic synthesis, and illustrated to show consistency of the West African and Laurentian reconstructions in a plausible global-scale paleogeographic context.

Volcanic rocks, such as those found in the Ouarzazate Group, record pinpointed variation of the magnetic field over time. However, this record may be sporadic or discontinuous because of the discrete nature of their eruptive history. By contrast, magnetizations of hematite within sedimentary rocks are acquired slowly through multiple processes during deposition and diagenesis, providing a time-averaged record of the ancient magnetic field. If the magnetic field retains an underlying time-averaged GAD geometry, its mean direction will be reflected in these magnetizations, and shorter-lived excursions may be smoothed out or altogether absent (*22*). Thus, combining magnetic directions from similarly aged volcanic and sedimentary rocks may reveal both the instantaneous variability of the magnetic field and its time-averaged axial component. Data from the redbed section BA07 produce a paleomagnetic pole at (16.7°N, 012.4°E), which would correspond to a paleolatitude of 67.6° if the time-averaged geomagnetic field was purely dipolar and aligned with the planetary spin axis. The results from the three volcanic sections (BA01, BA04, and BA08) yield a paleomagnetic pole at (25.7°N, 023.8°E). Because the igneous dataset is strongly streaked, which we interpret as recording enhanced and directionally biased (paleolongitudinally aligned) PSV of the Ediacaran geodynamo, Bingham rather than Fisher statistics are most appropriate for defining uncertainty bounds on this estimate (*23*, *24*). The overlapping error ellipses of the data from the red beds and volcanic rocks (Fig. 3) suggest that (i) the time-averaged geomagnetic field was stable despite the enhanced PSV, and (ii) our magnetostratigraphic sampling of volcanic cooling units, emplaced over ~1-Ma duration, adequately documents the mean geomagnetic state. The near-polar paleolatitude implied from our high-inclination remanences in both sedimentary and volcanic rocks is consistent with indications of glaciogenic activity (*20*) in the same measured sections (Fig. 1), likely as part of a regional rather than panglacial ice age (*25*–*27*), and it provides further support for parallelism of the geodynamo mean state with the planetary spin axis.

## DISCUSSION

Many Ediacaran paleomagnetic datasets from igneous rocks on other cratons show similar patterns of remanence distributed along great circles (*1*). In Laurentia, paleomagnetic directions from the ~565 Ma Sept-Iles composite intrusion may result from incomplete separation of ancient remanence with variable viscous (recent) overprints (*13*). Paleomagnetic data from the ~570 to 550 Ma Catoctin Formation are also distributed along a great circle and have been grouped into shallow and steep directions previously interpreted as resulting from two independent ages of remanence (*28*). In western Laurentia, hematitic sedimentary rocks of the Johnnie Formation contain a record of the Shuram negative carbon isotope excursion that is globally dated to ~574 to 567 Ma (*29*) and therefore coeval with the Catoctin record. As with the Ouarzazate red beds of the present study, the Johnnie paleomagnetic data are unstreaked (*10*). We calculate a Johnnie Formation mean paleomagnetic result using the same statistical treatment as that of section BA07 in the present study, yielding a pole at (09.7°N, 351.2°E) that we interpret as an accurate representation of the time-averaged geodynamo axis aligned with the planetary spin axis (fig. S6). Treating the Catoctin Formation data in a similar procedure to that used for the Ouarzazate volcanic sections, the Laurentia mean pole at (20.3°N, 343.6°E) has a Bingham uncertainty ellipse that overlaps the Johnnie pole and its error oval (Fig. 3).

West Africa and Laurentia are the only two cratons globally that contain coeval sedimentary and volcanic records from ~570 to 565 Ma; they provide complementary, yet consistent, estimates of the paleomagnetic pole when treated with Fisher (or Kent) and Bingham statistics, respectively. We generate a paleogeographic reconstruction of the two cratons in a global context (Fig. 3): assigning the compaction-corrected sedimentary poles to the spin axis according to standard paleomagnetic practice but, additionally, aligning the long axes of the Bingham ellipses from volcanic rocks on the assumption that PSV occurred along globally consistent paleolongitudes. Such consistency could result from a persistent equatorial geomagnetic dipole (*2*, *5*) and/or long-wavelength mantle structure dictating heat flow variability across the core-mantle boundary (*30*, *31*). The paleogeographic reconstruction generated by this method (Fig. 3) is plausible in the context of Gondwanaland amalgamation and late Ediacaran to Cambrian opening of the Iapetus Ocean (*5*). For future reconstructions of late Ediacaran cratons that lack coeval volcanic and sedimentary paleomagnetic records, we propose that either a Fisher, or Kent when an inclination correction is appropriate, statistical treatment of sedimentary data or a Bingham analysis of volcanic data can produce reliable estimates of the planetary rotation axis. Further application of our hypothesis through the latter treatment can also generate paleolongitude estimates, representing a breakthrough in deep-time paleogeographic reconstructions.

A nonuniformitarian late Ediacaran geomagnetic field with enhanced PSV along consistent paleolongitude bands, or a persistent equatorial dipole component varying in strength, or both of these factors, may be unique in Earth’s history. Paleointensity measurements of late Ediacaran rocks are anomalously weak (*11*, *32*) and consistent with models that predict a weak and unstable field preceding inner core nucleation (*33*–*35*). Alternatively, the exceptionally enhanced PSV illustrated by Ouarzazate paleomagnetic data and coeval igneous records could represent an extreme example of a recurring geodynamo feature at ~200 Ma intervals. Both the Late Jurassic and Devonian magnetostratigraphic records indicate rapid reversals and weak paleointensities, similar to the hyperactivity of the Ediacaran (*36*–*38*). Paleogeographic reconstructions from these three intervals of time are controversial because of the highly scattered paleomagnetic poles and have led to proposals of rapid TPW and other explanations (*39*–*41*). Rapid, oscillatory TPW has also been proposed for the Ediacaran (*12*, *14*) based on some of the same large great-circle dispersions of remanence directions considered above. Enhanced PSV can be regarded as an alternative explanation to TPW in these datasets (*5*). If any significant TPW did occur in late Ediacaran time, it should be documented by directional variability across continuous magnetostratigraphic sections of sedimentary rocks [e.g., (*42*)], which are more successful in averaging the enhanced PSV that dominates igneous-based datasets from that period. Continued paleomagnetic study of Ediacaran successions globally will create a high-resolution record of PSV and determine the relationship, if any, between TPW and the paleomagnetic record during the Ediacaran.

## MATERIALS AND METHODS

Paleomagnetic samples were taken in the field using a gasoline-powered drill and oriented using a magnetic and sun compass. Measurements were made at Yale University using a 2G Enterprises DC SQuID magnetometer and automatic sample changer (*43*). High-resolution stepwise thermal demagnetization was used to isolate characteristic magnetizations. Up to 30 temperature steps were used to demagnetize samples to 685°C by increments as low as 3°C. Magnetic directions were calculated using principal components analysis (*44*). Statistics were analyzed using the Python package PmagPy (*45*).

Volcanic samples were taken stratigraphically in groups of eight and grouped by flow unit. A Fisher mean was calculated for each flow and then converted to a virtual geomagnetic pole (VGP). Eigenvectors were then calculated from the distribution of VGPs, with the two principal moments yielding a 95% Bingham statistical error ellipse. In contrast to Fisher statistics, the Bingham distribution caters to asymmetrical variance about the mean. Although Bingham statistics are most commonly used in paleomagnetic studies for axial (bipolar) distributions, the function is equally well suited to describe an elongated distribution of unimodal vectors.

Sedimentary samples were taken every half meter throughout section BA07. Magnetizations from red beds often contain multiple generations of hematite. A postdepositional remanent magnetization is acquired when magnetic grains within the sediment pile rotate to align with the geomagnetic field. These grains can continue to rotate within the sedimentary pile for thousands of years after burial, effectively averaging secular variation (*46*). Magnetizations in red beds can also hold a chemical remanent magnetization, formed within pore spaces after deposition. This hematite is acquired slowly as iron-bearing silicates weather, liberating iron that reprecipitates as intrastratal hematite (*47*). This protracted process is able to “smooth” PSV. We correct compaction-induced inclination shallowing, a well-known complication of fluvial sediments, by following the procedure outlined in the supplement of Pierce *et al.* (*48*). A more robust statistical treatment is not feasible in our case as these methods require a very large dataset that is unattainable in section BA07 because of its limited spatial extent and thickness. The method in Pierce *et al.* (*48*) resamples a compilation of flattening factors drawn from published studies of hematite-rich rocks to unflatten paleomagnetic directions. Compared to implementing a single flattening correction, this method more appropriately represents the uncertainty associated when correcting inclination shallowing. A Kent ellipse is then defined that encloses 95% of the mean directions. Several methods were compared for correcting compaction-induced shallowing, and the outcome of the study and our conclusions were unaffected by such details (fig. S11).

The poles from the volcanic rocks and red beds can then be compared. The similarity of these poles (Fig. 3) provides confidence that the means represent the axial component of the geomagnetic field. This method was then extended to data from Laurentia and a previous dataset from Morocco, yielding consistent results in both cases. Using the GPlates reconstruction software (*49*), these poles were then used to create a paleogeographic reconstruction by overlapping the ellipses from the datasets.

### University of Geneva geochronology methods

Zircon crystals were separated using standard techniques (crushing, milling, and sieving to <300 μm, followed by density separation using a Wilfley Table, Frantz magnetic separation, and methylene iodide heavy-liquid separation). Zircon U-Pb ages were determined following the standardized CA– isotope dilution (ID)–TIMS technique (*50*, *51*). Single zircon crystals (euhedral, needle-shaped acicular, long- or short-prismatic) were handpicked and transferred into quartz crucibles for thermal annealing at 900°C for 48 hours and subsequent chemical abrasion at 210°C for 12 hours, following chemical abrasion technique of Mattinson (*50*). After chemical abrasion, the leachate was pipetted out completely, and the zircons were rinsed in water and flushed overnight in 6 M HCl on the hot plate at 80°C. Afterward, the zircons were ultrasonically cleaned four times for about 15 min each time, alternating water and weak HNO_3_ acid washing. Each single zircon grain was loaded for dissolution into precleaned Savillex capsules, spiked with 4 to 6 mg of the EARTHTIME ^202^Pb-^205^Pb-^233^U-^235^U tracer solution (*51*, *52*) and dissolved in 70 μl of hydrofluoric acid (HF) and traces of HNO_3_ at 210°C for about 60 hours in Parr bombs. After evaporation, the residue was redissolved overnight in 25 μl of 6 M HCl. Samples were then dried down again and redissolved in 80 μl of 3 M HCl. Lead and uranium were separated by anion exchange chromatography (*53*) in 40-μl columns using ultrapure HCl and H_2_O, and finally dried down with 3 μl of 0.01 M H_3_PO_4_.

The isotopic analyses were performed at the University of Geneva on a TRITON mass spectrometer equipped with a MasCom discrete dynode electron multiplier. The linearity of the multiplier was calibrated using U500, Sr SRM987, and Pb SRM982 and SRM983 solutions. The dead time was determined to be constant at 22.5 ns for up to 1.3 Mcps and at a Faraday/SEM yield between 93 and 94%. Isobaric interferences from BaPO2^+^ and Tl^+^ were monitored on masses 201 and 203, and since no statistically significant signal was observed on the controlled masses, no correction was applied. Lead isotopic fractionation was corrected on the basis of the certified value of ^202^Pb/^205^Pb = 0.99924 ± 0.03%, 1σ of the EARTHTIME ^202^Pb-^205^Pb-^233^U-^235^U tracer. The U mass fractionation for the same analyses was calculated using the ^233^U–^235^U ratio of the double spike solution (0.99506 ± 0.01%, 1σ). The average Pb and U fractionation factors determined by EARTHTIME ^202^Pb-^205^Pb-^233^U-^235^U tracer were 0.15 ± 0.02%/amu and 0.10 ± 0.02%/amu (1σ) respectively. Both lead and uranium were loaded with 1 μl of silica gel–phosphoric acid mixture [modified after (*54*)] on outgassed single Re filaments. Lead isotope compositions were measured on the electron multiplier, while U (as UO_2_) isotopic measurements were made in static Faraday mode (using amplifiers equipped with 1013 ohm resistors) or, in case of insufficient U beam size, on the electron multiplier. Isobaric interference of ^233^U^18^O^16^O on ^235^U^16^O^16^O was corrected using a ^18^O/^16^O ratio of 0.00205 ± 0.9%. The measured uranium isotopic ratios were corrected assuming a sample ^238^U/^235^U ratio of 137.818 ± 0.045 [2σ, (*55*)]. All common Pb in the zircon analyses was attributed to the procedural blank. Over the course of this study, 15 total procedural blanks were measured, yielding the following blank isotopic composition: ^206^Pb/^204^Pb = 17.10 ± 1.2, ^207^Pb/^204^Pb = 15.07 ± 0.7, ^208^Pb/^204^Pb = 36.17 ± 0.7 (1-sigma %). Uranium blanks are <0.1 pg and a value of 0.05 pg ± 50% was used in all data reduction.

The initial statistics, data reduction, and age calculation were done using the TRIPOLI and Redux software (*56*). All ^206^Pb/^238^U and ^207^Pb/^206^Pb ratios were corrected for initial disequilibrium in ^230^Th/^238^U using Th/U [magma] = 3.5 ± 1 (1σ). The accuracy of the measured data was assessed by repeated analysis of the 100-Ma synthetic solution (*57*), yielding an internal reproducibility in ^206^Pb/^238^U dates of better than 0.05%. The 100-Ma synthetic solution measured during the period of zircon analyses with EARTHTIME ^202^Pb-^205^Pb-^233^U-^235^U tracer yielded mean ^206^Pb/^238^U = 100.175 ± 0.012/0.029/0.11 Ma [mean square weighted deviation (MSWD) = 1.4, *n* = 8]. All uncertainties reported are at the 2 sigma level, following x/y/z systematic of (*58*), whereby x = internal uncertainty, y = added systematic uncertainty, and z = added uncertainty of the decay constants. All data are reported in the table S2, with internal errors only, including counting statistics, uncertainties in correcting for mass discrimination, and the uncertainty in the common (blank) Pb composition. The MSWD value of weighted mean is within the range of acceptable values at 95% confidence level and for n-1 degrees of freedom, defined in (*59*). Some of the analyses are slightly inversely discordant (−0.13 to −0.29%) but still within the uncertainty of the decay constants. The reason for this very likely lies in the interplay between (i) uncertainty of ^207^Pb/^204^Pb ratio of the blank (influencing measurements with lower Pb*/Pbc ratio) and (ii) the fact that all samples were corrected for the tracer contribution without correction of the minor isotope composition of the tracer itself (usually the analyses with the lower blank are strongly affected by this).

### ETH Zurich geochronology methods

Selected zircons from sample RA03 were analyzed for high-precision geochronology using CA-ID-TIMS at ETH Zurich. The crystals were annealed at 900°C for 60 hours and subsequently loaded in Teflon microcapsules for chemical abrasion with concentrated HF and placed in a high-pressure Parr bomb at 190°C for 12 hours. Afterward, the zircons were moved to Teflon beakers and washed in 6 M HCl on a hot plate (at 80°C) and in 4 M HNO_3_ in an ultrasonic bath. The crystals were then loaded back into the precleaned microcapsules for dissolution with concentrated HF and trace HNO_3_. The EARTHTIME ^202^Pb-^205^Pb-^233^U-^235^U isotope tracer solution [ET2535; (*51*, *52*)] was added at this step to ensure complete sample-spike equilibration. The zircons were dissolved in a Parr bomb for 60 hours at 220°C.

The solutions were subsequently dried down and redissolved in 6 M HCl at 190°C for 4 to 6 hours in the Parr bomb. Then, the solutions were dried down and redissolved in 3 M HCl for column chemistry [modified procedure after (*53*)], which efficiently separates Pb and U isotopes from other elements. Uranium and Pb were collected and dried down with one drop of 0.02 M H_3_PO_4_. Finally, the samples were loaded onto outgassed zone-refined Re filaments with a silica gel emitter [modified from (*54*)].

Analyses were conducted on a Thermo Fisher Scientific TRITON Plus thermal ionization mass spectrometer equipped with an axial secondary electron multiplier and Faraday cups connected to 1013-ohm amplifiers in a setup described in von Quadt *et al.* (*60*) and Wotzlaw *et al.* (*61*). Lead isotopes and U oxides were analyzed using static multicollection in the Faraday cups, except for ^204^Pb which is analyzed in the axial secondary electron multiplier. Pb and U mass fractionation were corrected using the double spike (EARTHTIME ET2535) and assuming a sample ^238^U/^235^U ratio of 137.818 ± 0.045 [2σ; (*55*)]. Data reduction and visualization was performed using Tripoli and U-Pb Redux software (*56*). ^206^Pb/^238^U dates were corrected for initial ^230^Th/^238^U disequilibrium using a constant Th-U partition coefficient ratio of 0.2. All dates are reported with 2 sigma uncertainty, which are presented as μ ± x | y | z, where μ is the date, x is the uncertainty including only analytical uncertainties, y includes the uncertainty of the tracer calibration, and z also includes the uncertainty on the decay constant.

Bayesian age-depth modeling was conducted using Chron.jl (*62*), which implements a Markov chain Monte Carlo age-depth model in the Julia programming language (*63*). Since the Chron.jl age-depth model does not make any assumptions about the steadiness or continuity of sedimentation rate, it is potentially suitable for use in sections where sedimentation is not necessarily expected to be regular and continuous but rather flashy and discontinuous—including clastic sedimentary and volcanic sections such as those of the Ouarzazate supergroup. As inputs to this model, we provided the three Gaussian age constraints from ID-TIMS geochronology (i.e., 567.937 ± 0.035 Ma for RA03 at a height of 2 m, 566.78 ± 0.19 Ma for MR34 at a height of 97.8 m, and 562.220 ± 0.079 Ma for MR41 at a height of 445.9 m, excluding decay constant and tracer uncertainties) and the measured stratigraphy from the combined P22-BA-01 and P22-BA-02 sections, particularly including the position of observed unit boundaries between distinct volcanic flows and ignimbrites, which were treated as potential hiatuses in the age-depth model. Because of the coherence of the lowest [N] paleomagnetic samples from the bottom 73.8 m of the measured section, this portion of the stratigraphy was treated as geologically instantaneous and thus reduced to zero thickness for the purposes of age-depth modeling. The model was run with a vertical resolution of 0.1 m, collecting 30,000 accepted proposals from the stationary distribution with a sieving factor of 7444 (i.e., collecting only every 7444th accepted proposal from the stationary distribution to reduce autocorrelation) after discarding 74,440,000 steps of burn-in. The sieved post–burn-in log-likelihood distribution is shown in fig. S10. All scripts used to run this model and plot the results are provided in the Supplementary Materials.

## Supplementary Materials

### The PDF file includes:

Supplementary Text Figs. S1 to S11 Legends for tables S1 to S4 Legends for data S1 and S2 References
