# Supplementary material for: Magnetostratigraphic constraints on the late Ediacaran paleomagnetic enigma
Source: Sci Adv. 2025 Oct 3;11(40):eady3258. doi: 10.1126/sciadv.ady3258 (PMC12494014; doi:10.1126/sciadv.ady3258)
Supplement: Supplementary file 1 — Supplementary Text Figs. S1 to S11 Legends for tables S1 to S4 Legends for data S1 and S2 References [file sciadv.ady3258_sm.pdf]

Science Advances
AAAS

Supplementary Materials for

Magnetostratigraphic constraints on the late Ediacaran paleomagnetic enigma

James S. Pierce *et al.*

Corresponding author: James S. Pierce, james.pierce@yale.edu

Sci. Adv. **11**, eady3258 (2025)
DOI: 10.1126/sciadv.ady3258

The PDF file includes:

Supplementary Text
Figs. S1 to S11
Legends for tables S1 to S4
Legends for data S1 and S2
References

Other Supplementary Material for this manuscript includes the following:

Tables S1 to S4
Data S1 and S2

Supplementary Text

Regional Geologic Context

The Ouarzazate Group was emplaced at the northwest margin of the West African Craton (WAC) between 580 Ma and 540 Ma. It experienced only limited, late-stage Pan-African deformation and was deposited on a highly variable basement topography, which, coupled with the large and rapid variations in thickness of the Ouarzazate Group itself, strongly suggests that this group was deposited during extensional/transtensional movement (*19* and references therein). The Ouarzazate Group covered a gigantic area of ~2 × 106 km2, with an estimated magma volume of ~1 × 106 km3 and volcanic thicknesses more than 2000 m. This huge late Neoproterozoic (Ediacaran) magmatic event (the so called Ouarzazate Event or Central Iapetus Magmatic Province labelled CIMP, *67*) was related to intense hydrothermal activity, as exemplified by the main deposits of Imiter and Zgounder (Ag-Hg), Bou Azzer (Co-Ni-As-Ag-Au), Iourirn (Au), and Bou Madine (Cu-Pb-Zn- Au-Ag; *68-69*). The most complete and best-preserved sequences of these volcanic occurrences of the Ouarzazate Group are in the Ouarzazate–Siroua–Bou Azzer regions.

About ten Precambrian erosional inliers are located south of the Anti-Atlas Major Fault (Fig. S1), which is generally interpreted as a reactivated mid-Neoproterozoic bounding structure of the WAC (*70*). Regional folding took place during the Variscan/Hercynian orogeny and made the inliers dome-shaped (*71*). Metamorphism due to that event is stronger towards southwest but only attained lower greenschist grade at its maximum in the Anti-Atlas ranges (*72)*. The low grade of metamorphism permits retention of primary magnetization because the peak temperatures are well below the unblocking temperature of low-Ti magnetite that is the main carrier of remanence in mafic rocks. Margins of the inliers are gently tilted, as indicated by nonconformably overlying Ediacaran-Paleozoic sedimentary rocks, which bear the brunt of Variscan buckle-like deformation that leaves the basement largely intact (*73*).

A widespread magnetic overprint with a shallow, southeast direction produces paleomagnetic poles that overlap the 320-280 Ma interval of the Phanerozoic polar wander path for the WAC (*74*), corresponding to the Hercynian Orogeny and the Kiaman reverse geomagnetic superchron. This southeast and shallow magnetic direction partially overprints many of the samples in this study, but primary information is almost always retained.

Paleomagnetic Results from Bou Azzer (Extended Description)

Samples from volcanic rocks generally each contain three vector components of the natural remanent magnetization (NRM), isolated clearly by progressive thermal demagnetization (Fig. S3). The first component to be removed, typically below 200°C, is directed north-down, consistent with the present Earth field (PEF) at the study area. An intermediate unblocking-temperature component is removed between 530 and 580°C, consistent with magnetite or titanomagnetite as a magnetic carrier. Its directions streak from the PEF direction toward the southeast and shallow, suggesting a mix of PEF and the expected Hercynian overprint direction. Finally, at highest unblocking temperatures above 660°C (consistent with hematite as the remanence carrier), a residual component is observed to hold highly variable directions across the entire stratigraphic section, yet internally consistent within each paleomagnetic site (Fig. S4 B,C,E,F).

Paleomagnetic data from the red siliciclastic units (redbeds) of section BA07 contain two components of the NRM: a low-temperature component with a north-down direction identical to

the PEF as measured within the adjacent igneous rocks, and a high-temperature component unblocking in the range of 660-680°C that is directed steeply downward and southerly on average (Fig. S4D). A Fisher statistical mean of this ChRM component overlaps the site means from the uppermost igneous sites in the adjacent sections BA01 and BA08 (Fig. S4E), as well as the Bingham mean of all igneous sites from those sections (Fig. S4F).

Conglomerate Test

A cobble conglomerate with rounded rhyolitic clasts is encountered directly above geochronology sample MR41 (562.2 Ma) ~200m stratigraphically above sections BA01, BA07, and BA08. The conglomerate lies some hundreds of meters below the first appearance of Cambrian strata. Ten clasts were sampled, five of them multiply so with independently oriented drill cores. Characteristic remanence magnetizations (ChRMs) were isolated in the hematite unblocking temperature range, and yield pronounced scatter across the site. However, the multiply sampled clasts show internal consistency (Fig. S5). A Watson (*75*) statistical test shows that the clast dataset cannot be distinguished from a uniform ("random") distribution with any high degree of confidence. The result indicates that there is no pervasive regional overprint affecting hematite-bearing rocks of the Bou Azzer region.

Paleomagnetic Poles

As summarized in the Main text and Methods section, the significant trend of igneous ChRM directions across a small amount of time, combined with the conglomerate test implying primary magnetic remanence, suggests that between-site variability is likely due to geomagnetic paleosecular variation (PSV). That such variation is distributed along a great circle suggests an underlying structure to the PSV, which can be captured by Bingham statistics (*23, 24, 76*). Although Bingham statistics are commonly used in paleomagnetic studies to treat circularly symmetric bipolar datasets, their 3-axis eigenvector/eigenvalue calculation is equally amenable to strongly elongated unimodal distributions such as observed in the igneous data of this study. In contrast, redbeds from an immediately adjacent section BA07 yield a ChRM mean with a circularly symmetric distribution that can be treated with Fisher (*77*) statistics, or an inclination-corrected Kent distribution (48). Parameters for these means and others from the published literature are presented in Table S1.

Comparison to previous results from the Bou Azzer inlier

Uncertainty regarding the position of the WAC during the Ediacaran stems from a need for more high-quality data. Widely used criteria for assessing a paleomagnetic study's quality come from Van der Voo (*78*) and Meert et al. (*79*), which assign reliability values between one and seven, where a rating of four or higher is generally regarded as robust. The lone Precambrian paleopoles that satisfy more than four of these criteria come from Robert et al. (*14*). The authors published two poles: one that places the WAC at low latitude and one at high latitude pole. Robert et al. (*5*) have recently reported that the rocks yielding these two sets of directions are nearly indistinguishable in age and that the paleomagnetic data should represent anomalous geomagnetic field behavior. Both poles from Robert et al. (*14*) fall along a great circle that is similar to that reported in our study (Fig. S6). We reanalyzed the paleomagnetic data from Robert et al. (*14*) and calculated a Bingham mean for all data. We found that data from both studies follow similar geomagnetic field behavior and lend support for the use of our method during this interval of the Ediacaran.

Reanalysis of Ediacaran data from Laurentia

Some previous studies from Ediacaran Laurentian rocks show seemingly primary magnetizations but with perplexing results (*3, 6, 7*). At certain times, rocks that are indistinguishable in age have produced paleomagnetic directions that are irregularly distributed and suggest rates of apparent polar wander that are not easily explained by plate tectonics or true polar wander. This led to researchers choosing a subset of directions that they felt were the most reliable and/or may have represented the axial field. We reevaluated the results from those studies, incorporating all data rather than choosing subsets, led by our hypothesis that the geomagnetic field had greatly enhanced secular variation with a GAD bias over long timescales. We used Bingham statistics to determine directional means for these data to calculate paleopoles. In each of the three cases, VGPs fall along a great circle.

Fig. S1.

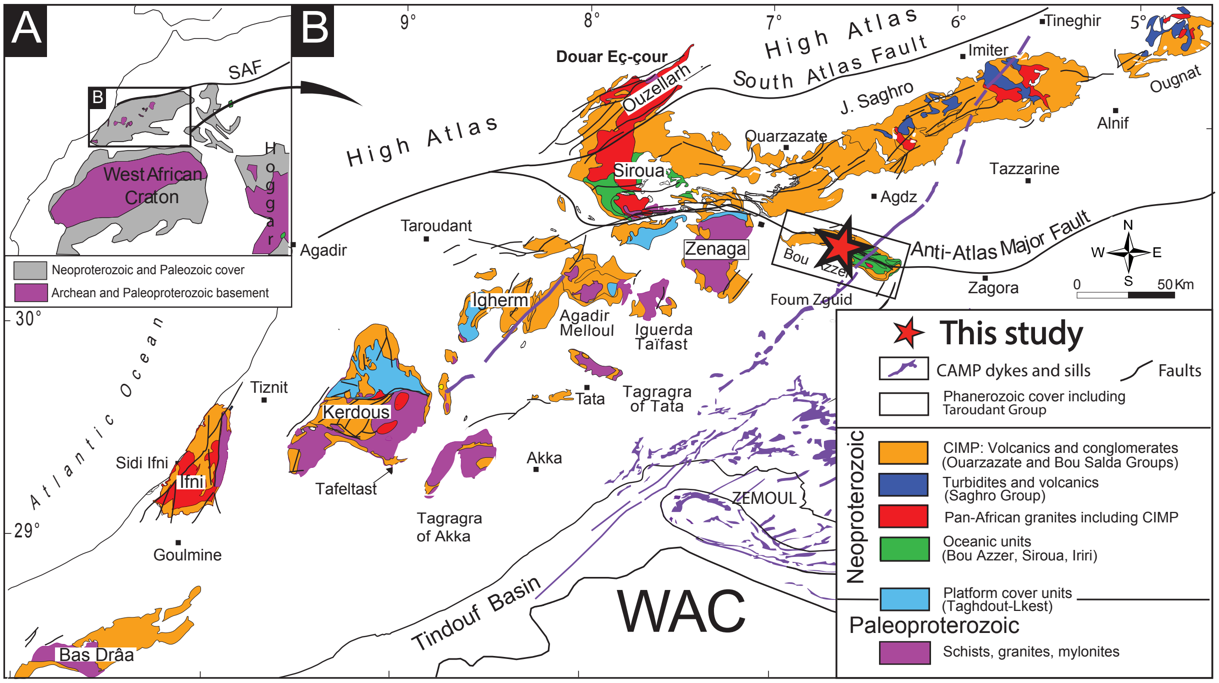

A map showing regional geologic context, emphasizing Neoproterozoic inliers. Inset (A) illustrates the South Atlas Fault (SAF) in relationship to the Anti-Atlas Mountains and the West African Craton (WAC). Panel (B) locates the Bou Azzer inlier (boxed) in context with the Anti-Atlas Major Fault, a Cryogenian suture. Rocks of the Ediacaran Ouarzazate Group, Central Iapetus Magmatic Province (CIMP, orange) straddle that suture and cover the entire WAC neoautochthon. After Gasquet et al. (*69*).

Fig. S2.

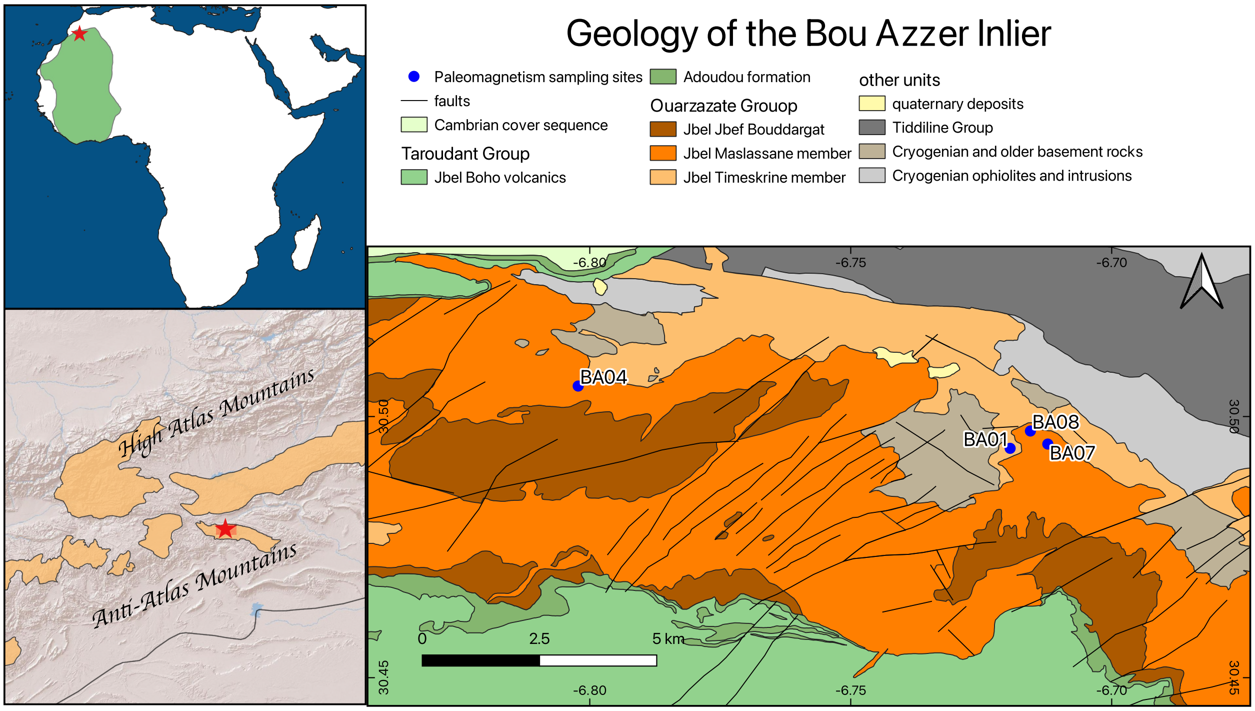

A map of the study area, northwest of the Bou Azzer inlier. Rocks here are gently dipping and younging to the south. Stratigraphic sections presented in this paper are marked BA01, BA07, and BA08.

Fig. S3A.

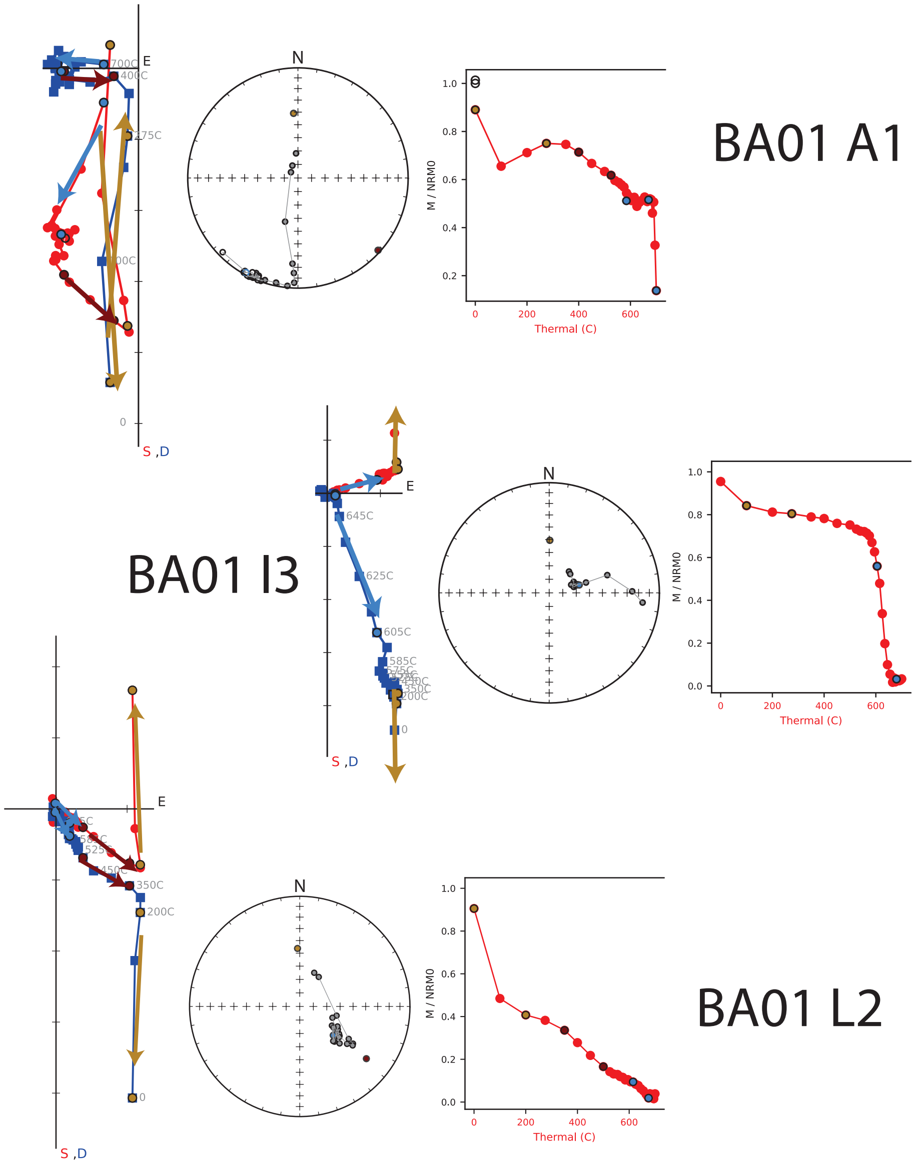

Shown are principal component analyses for a variety of representative samples from section BA01. Blue is the vertical axis, and red is the horizontal axis. Sample BA01 A1 has three distinct components including a well-defined mid-temperature component consistent with the expected Hercynian direction. Sample BA01, samples I3 and L2 have single component directions, that are commonly seen throughout the section.

Fig S3B.

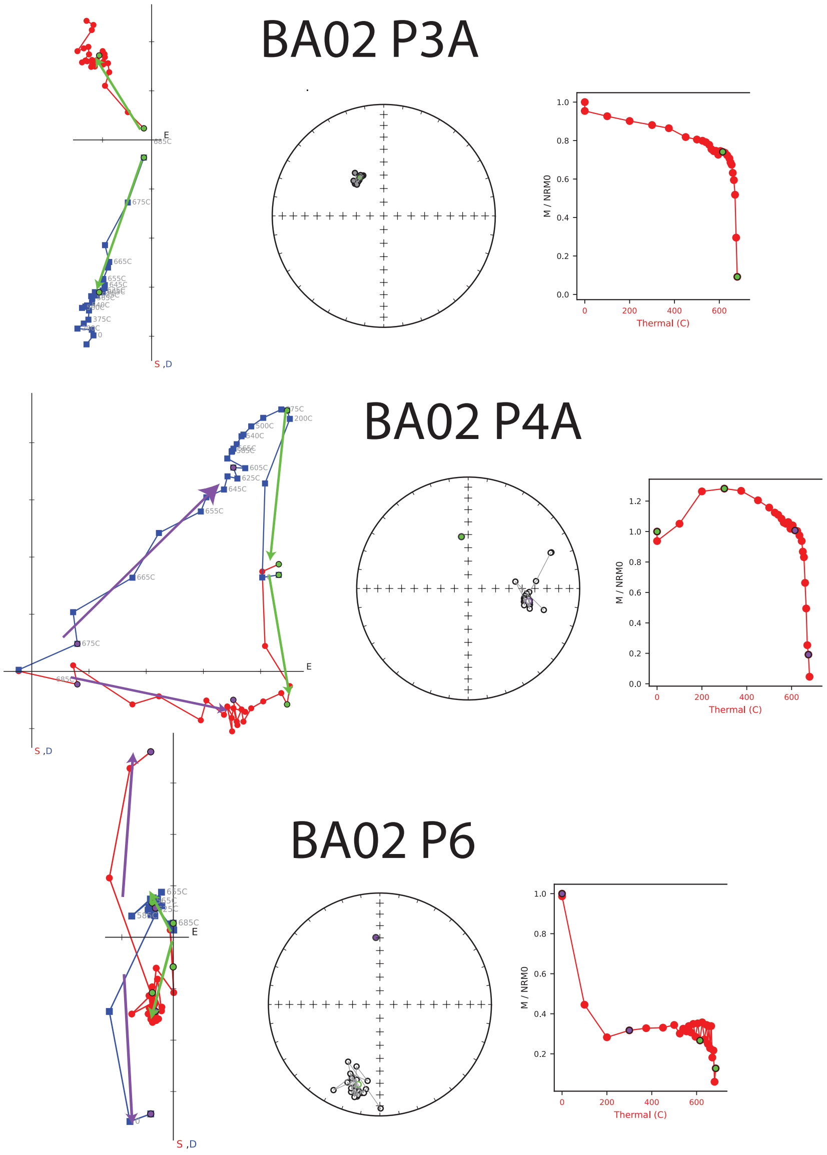

Results from the conglomerate test. Clasts of section BA02 site P show stable, predominantly single-component directions with unblocking temperatures similar to volcanic rocks in the other sections.

Fig S3C.

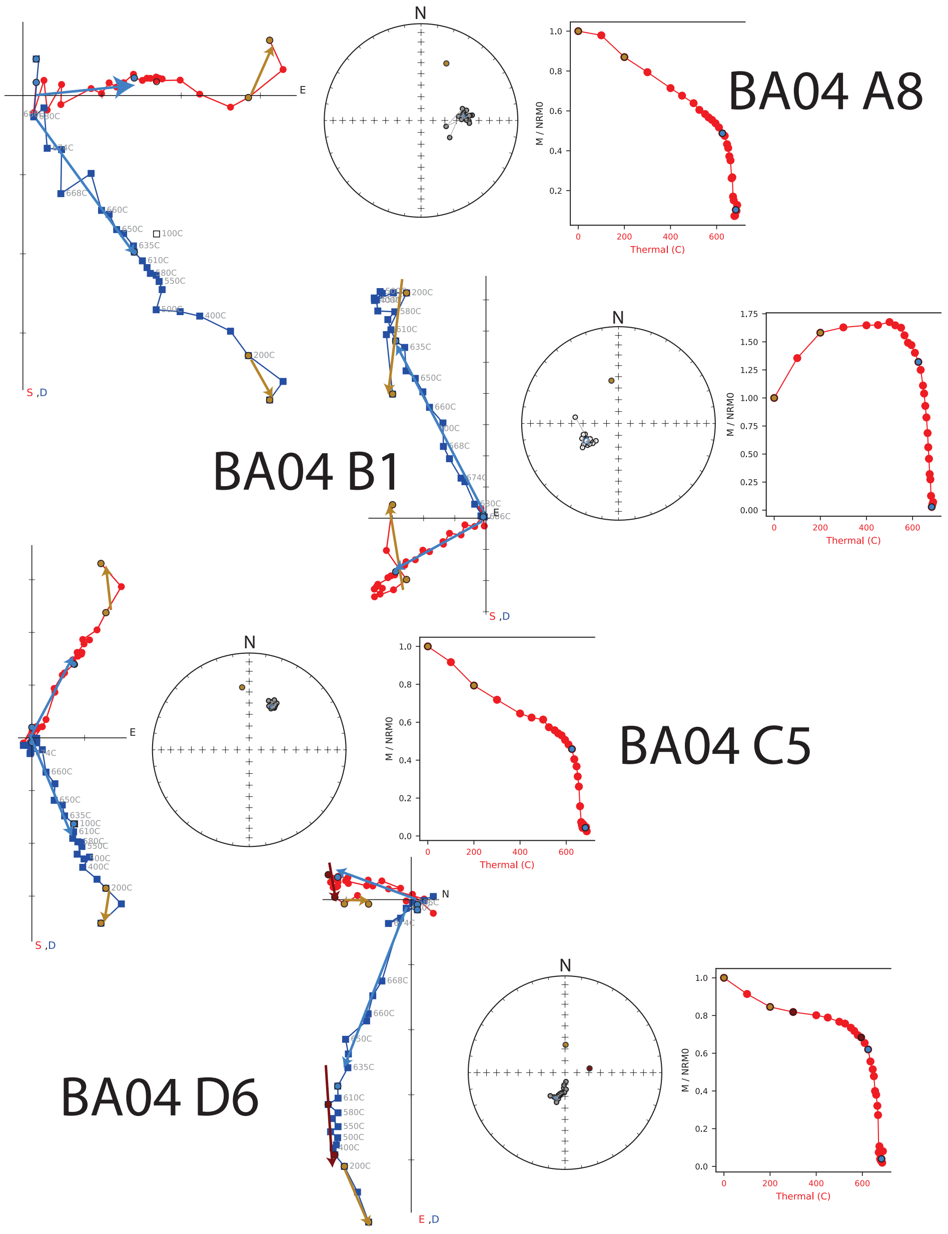

Results from section BA04 show stable, single-component directions.

Fig S3D.

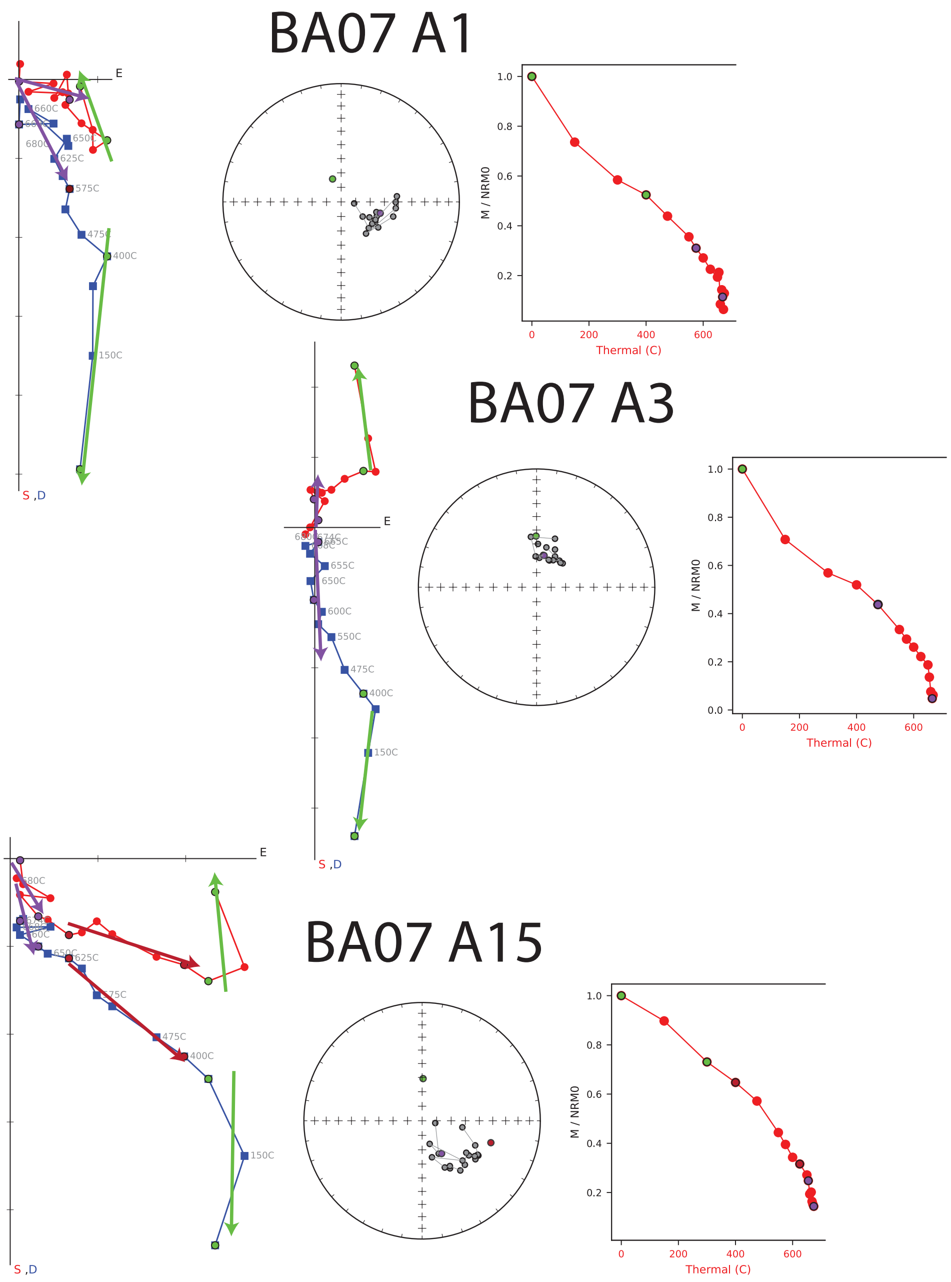

Results from redbeds of section BA07 contain steep high-temperature directions. Some samples retained a partial overprint with a lower unblocking temperature, such as with sample BA07 A15.

Fig S3E.

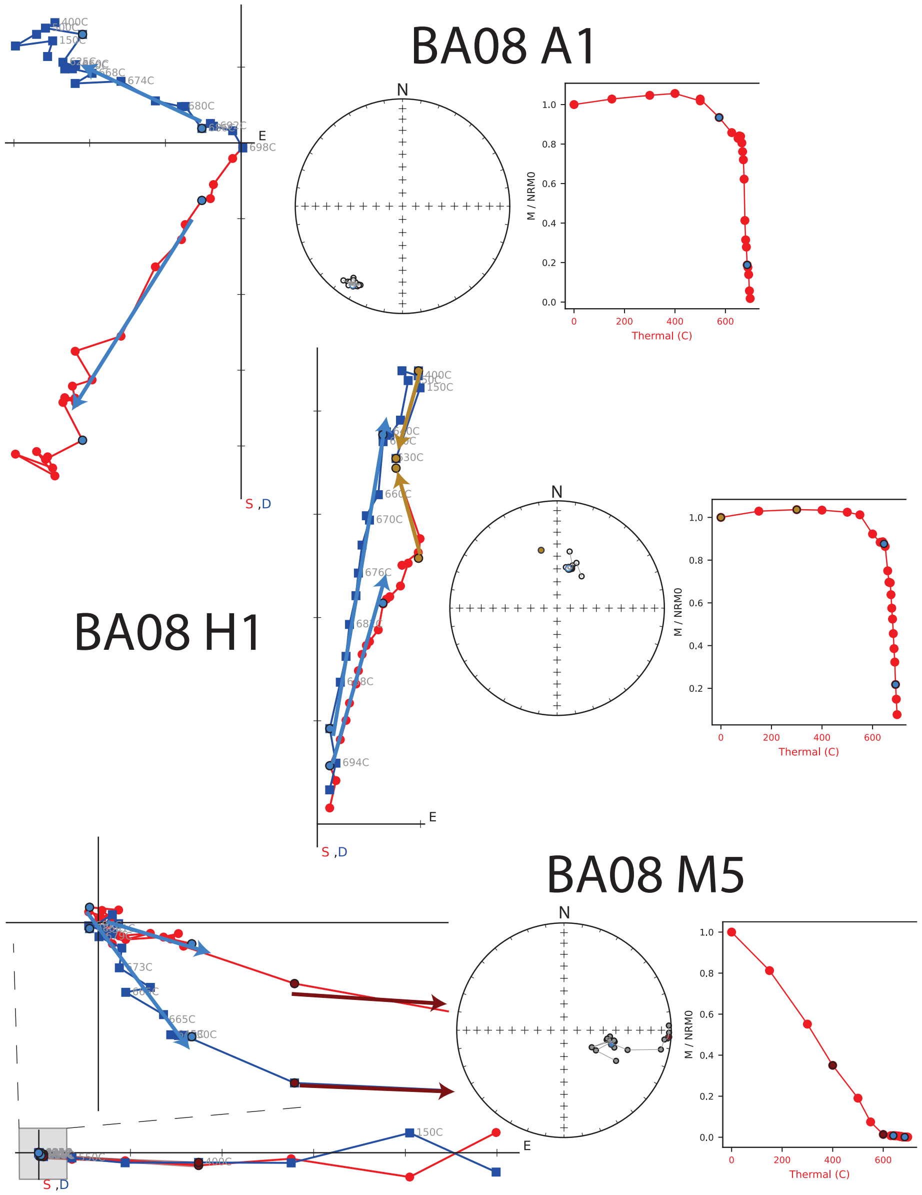

Magnetic directions from section BA08. These are similar to BA04, primarily having single-component directions. Sample BA08 M5, from near the top of the section, shows a significant overprint but retains a distinct, stable high-temperature direction.

Fig S4.

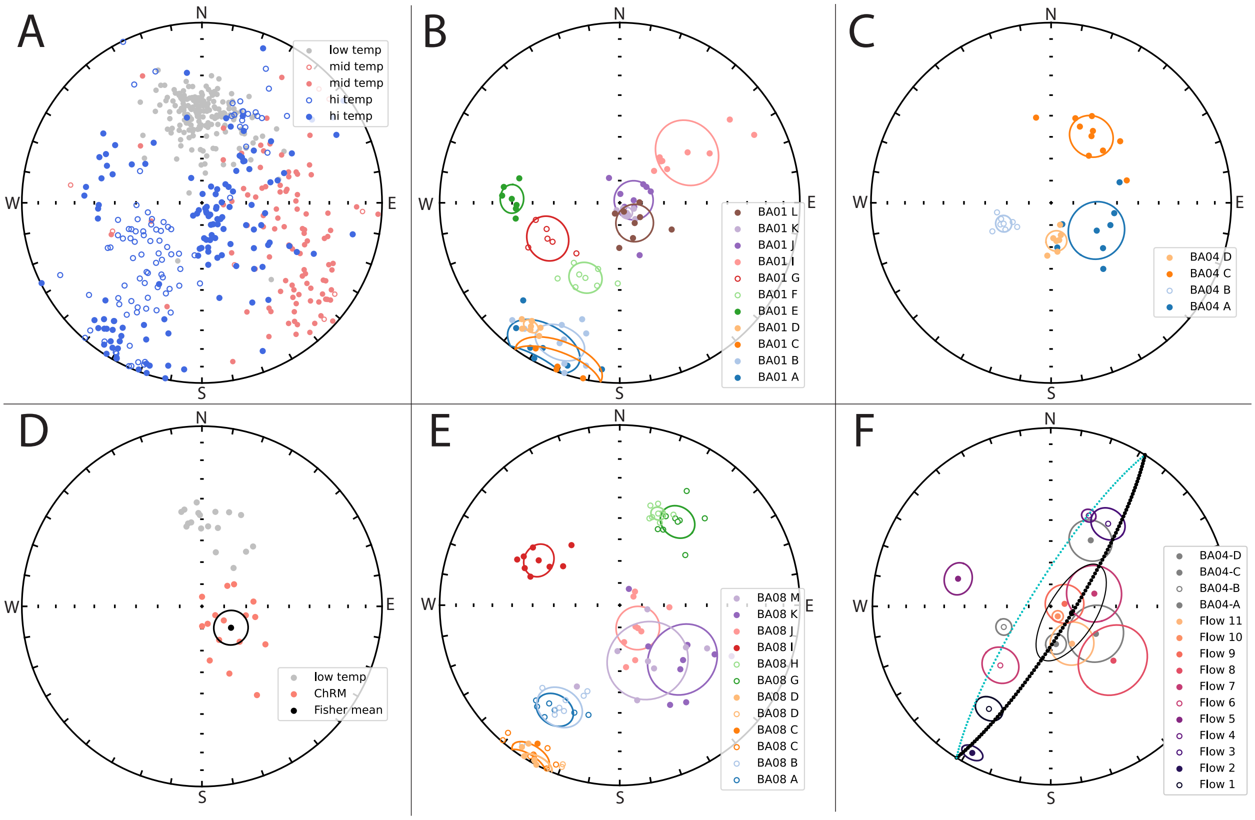

A summary of paleomagnetic directions plotted on equal area projections. A. All components in geographic coordinates from all volcanic rocks in this study. In grey color is a north-down low-temperature component; in coral color a moderate-temperature range (~530°-580° C) component; and in blue color is the set of components unblocking above 660°C. B. Paleomagnetic directions and site means from section BA01, showing internal consistency at each site and a trend from shallow to steep moving up section. C. Paleomagnetic directions and site means from section BA04. D. Paleomagnetic directions from section BA07, which consists of hematitic siliciclastic rocks. E. Paleomagnetic directions and site means from section BA08. F. Paleomagnetic directions from sections BA01 and BA08 were grouped based on lithostratigraphic and paleomagnetic correlations to avoid sampling bias. Site means from BA04 are plotted in grey, showing consistency with directions from other sections. A Bingham mean calculated from this dataset is superimposed along with a great circle along which the data is distributed.

Fig S5.

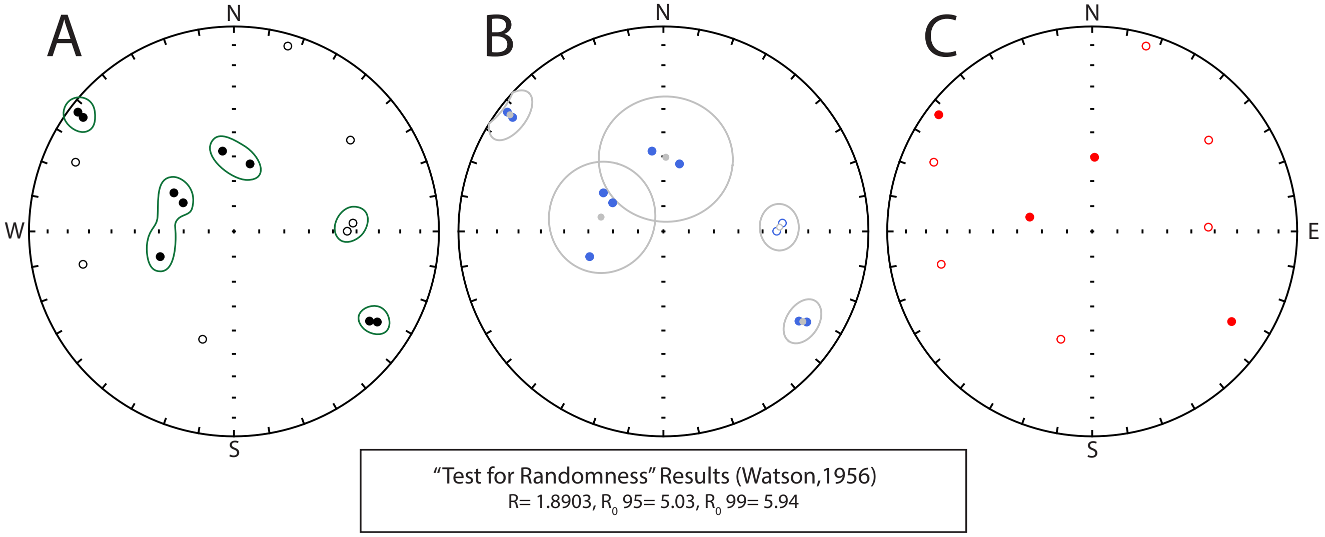

Analysis of the conglomerate test. A. All data from independently oriented cores. Five clasts were large enough to allow multiple sampling (green envelopes), which yielded consistent internal directions. B. Directional means calculated from each clast with multiple samples illustrate internal consistency (Fisher a95 circles shown). C. Using the mean directions from multiply sampled clasts, along with the single directions from the other clasts, a Watson common mean test (*75*) passes, with results shown in the box below the equal area plots.

Fig S6.

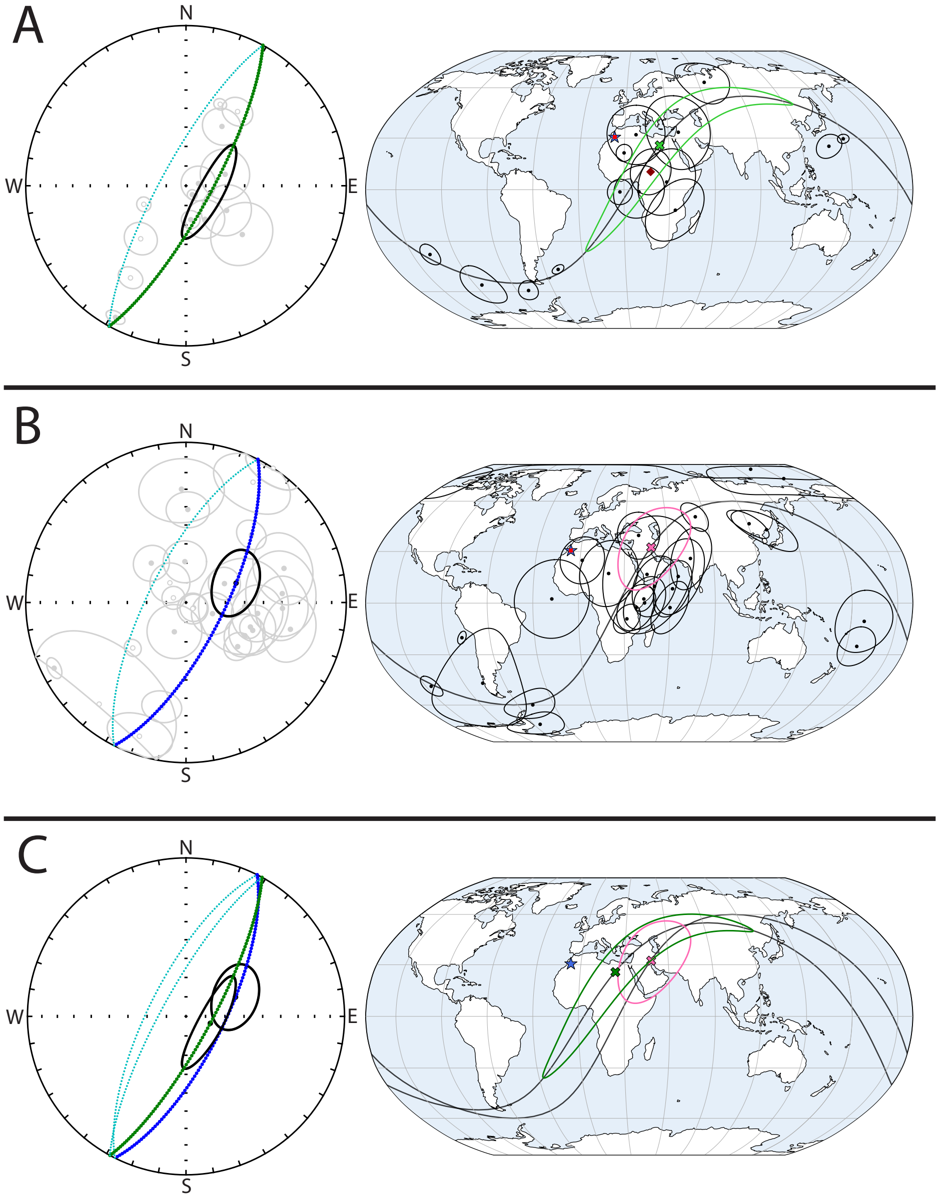

Comparison of VGPs from this study to Robert et al. (*14*). A. Paleomagnetic site means and overall Bingham ellipse from this study are plotted here on the left. On the right, virtual geomagnetic poles (VGPs) are plotted along with the Bingham mean and ellipse calculated using VGPs. B. The same treatment was applied to site means for components C and B1 by Robert et al. (*14*), showing a similar trend in data to our study. C. The Bingham ellipses and great circles from both studies are plotted together and show consistent distributions of directions.

Fig S7.

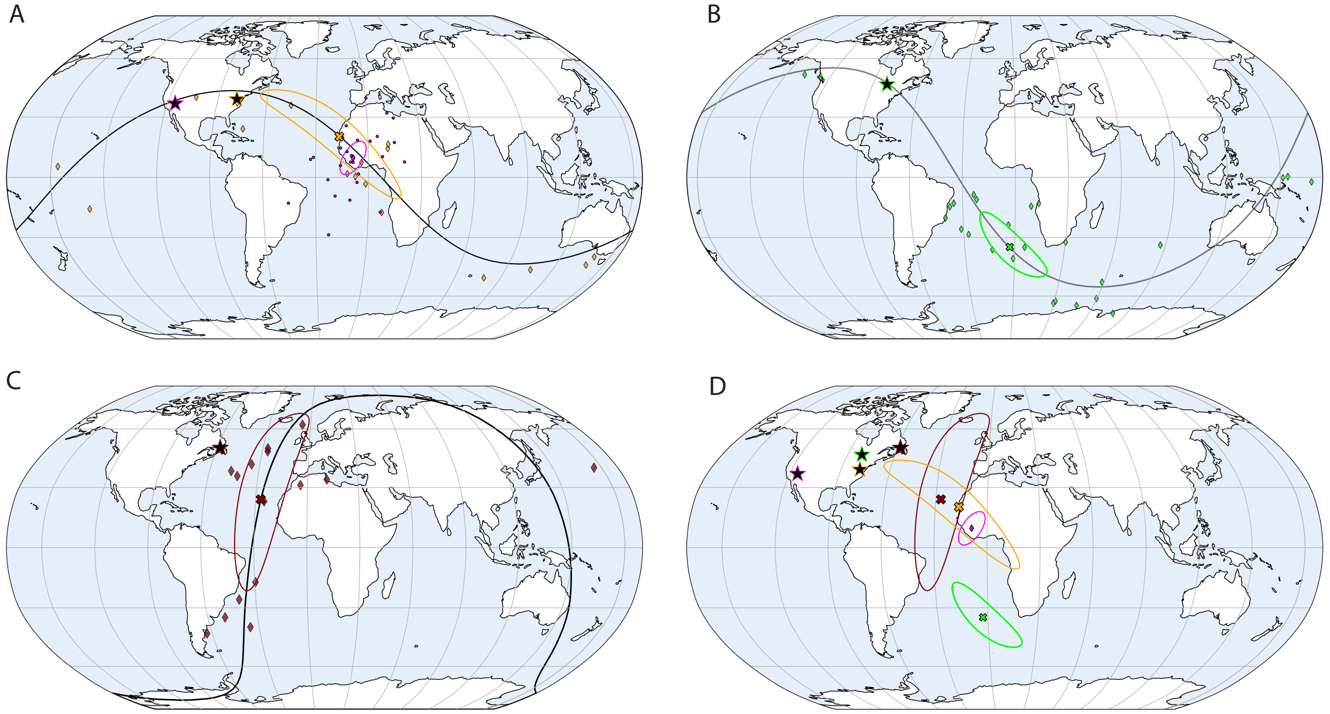

Application of the method presented in this paper to previously published studies of Ediacaran rocks of Laurentia. A. Plotted in orange is a Bingham mean ellipse calculated using data from ca. 570-Ma Catoctin igneous rocks in Meert et al. (*28*). The pole and dp/dm ellipse (derived from Fisher-distributed directions in local coordinates), plotted in fuchsia, were calculated using data from coeval Johnnie Formation siliciclastic rocks in Kodama (*10*). Data from the Johnnie Formation are corrected for inclination shallowing using f=0.6. However, using other methods, or omitting an inclination correction, does not change the conclusion of this study. The stars mark the sampling locations. These data were used to determine the placement of Laurentia relative to West Africa in our reconstruction (Figure 3). B. A Bingham mean is plotted using data from the ca. 587-585-Ma Grenville Dykes published in Halls et al. (*3*). C. A Bingham mean is calculated from the ca. 583-Ma Mutton Bay intrusion in McCausland et al. (*6*). D. Superposition of these selected late Ediacaran paleopoles from Laurentia with their Bingham uncertainty ellipses. Apparent polar wander of the axial dipole field is suggested for these studies, within the uncertainty ellipses shown.

Fig S8.

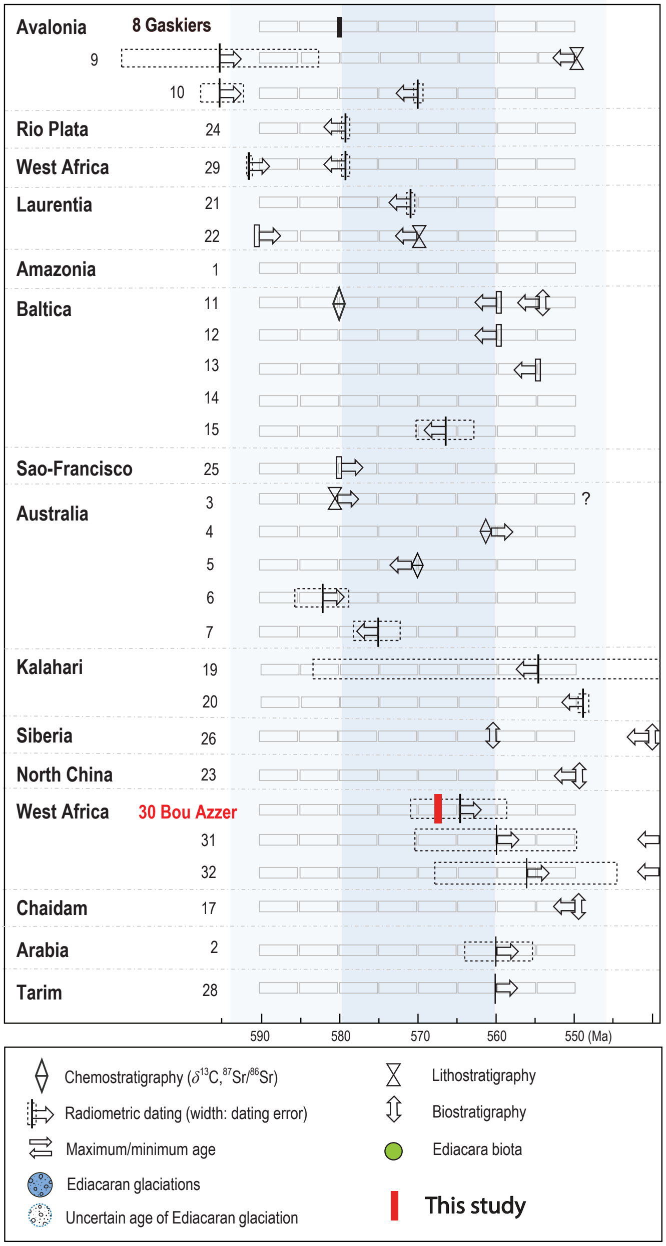

We find that the WAC is at high-latitude and supports regional Ediacaran glaciations. The red bars indicate precisely dated age constraints on glaciogenic units from Pu et al. (*80*) and our study. The West African Craton is at high latitude ~568-566 Ma. The numbers correspond to glaciogenic formations described in Table S1 from Wang et al. (*27*), where unit 30 represents the Bou Azzer glaciation. Figure modified after Wang et al. (*27*).

Fig S9.

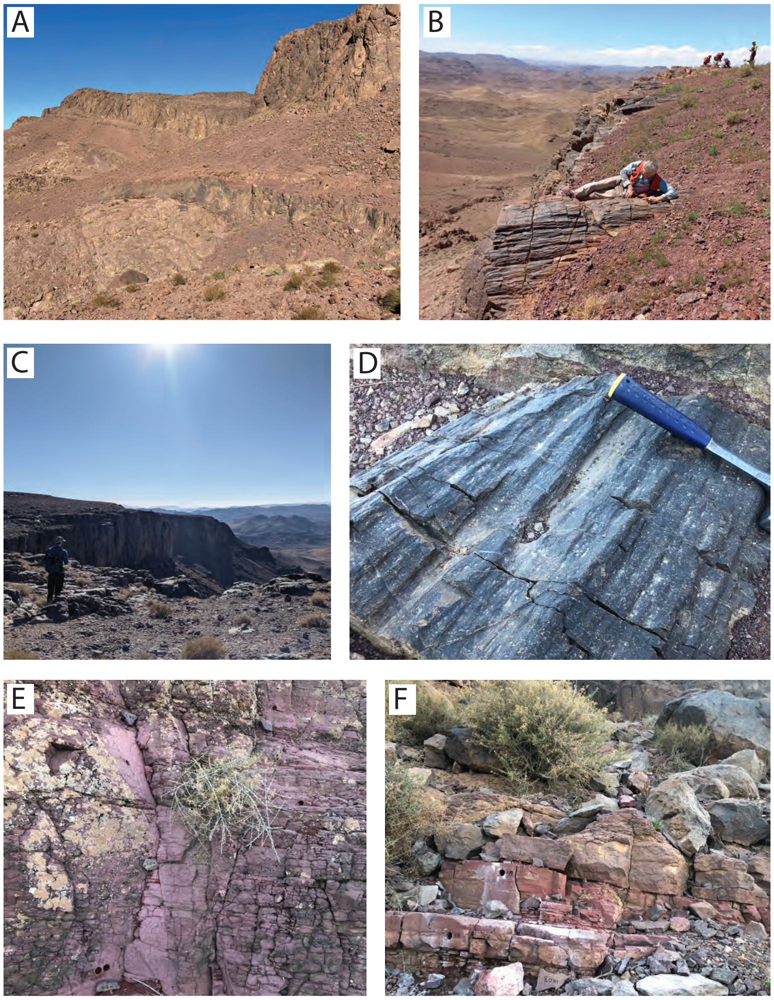

Section photos. A. Section BA01 with the cliff-forming rhyolite that yielded sample RA03 unconformably overlying Cryogenian rocks. B. Striated surface, purported to be of glacial origin by Vernhet et al. (*20*), which lies directly on layer that yielded geochronology sample MR34. C. The flat-lying fiamme ignimbrite marks the topographic high. In the background, looking south, are mafic volcanics of the Cambrian Jbel Boho. D. The scoured and grooved surface found in sections BA01 and BA08, described as glaciogenic by Vernhet et al. (*20*). E. The lowermost part of section BA07 containing red sandstone of variable grain size. F. The uppermost interval of section BA07 with fine-grained, silicified red sandstone.

Fig S10.

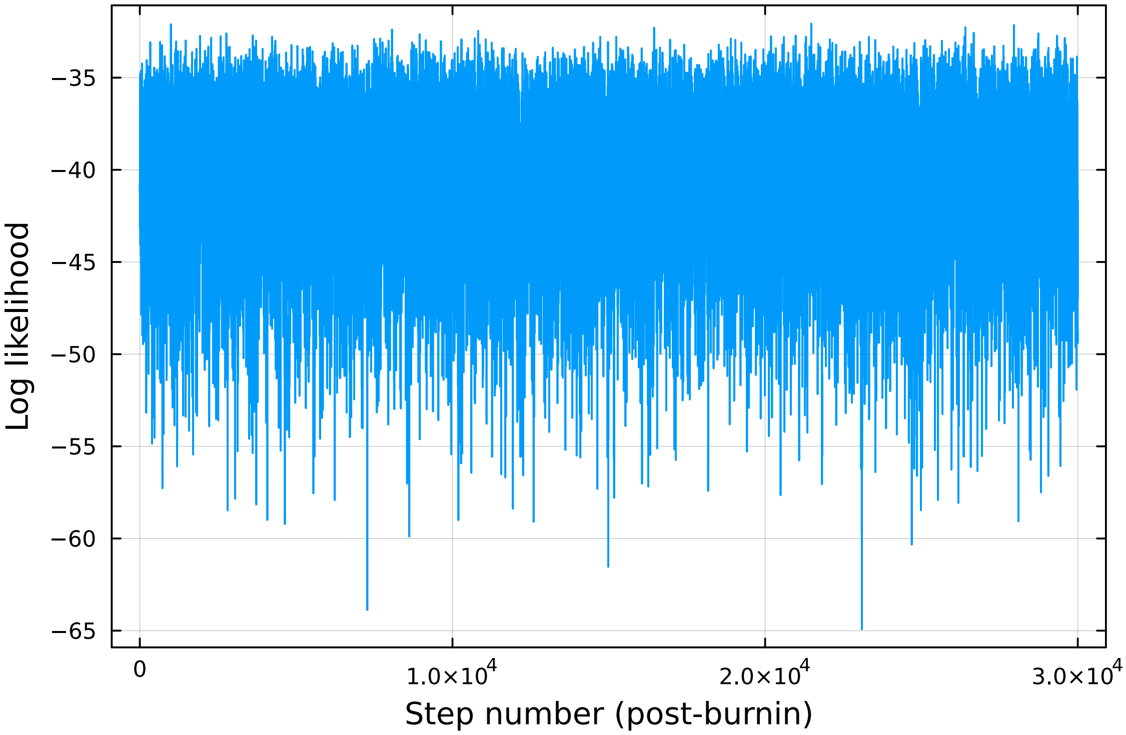

The sieved post-burnin log-likelihood distribution corresponding to the Bayesian age-depth model shown in Figure 2B, showing apparent stationarity.

Fig S11.

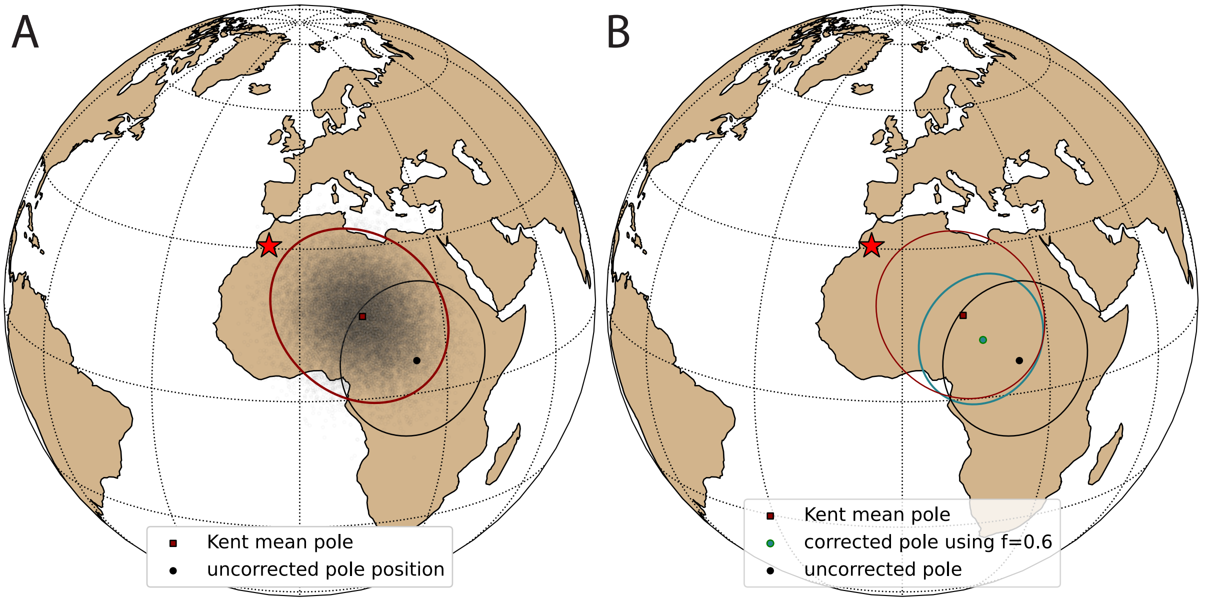

Comparison of different methods for correcting inclination shallowing by showing VGPs calculated from the sedimentary data of section BA07. A. The virtual geomagnetic pole for site BA07, uncorrected for compaction-induced inclination shallowing, is shown in black. The red ellipse is a Kent 95% confidence interval, fit to data corrected for inclination shallowing following the method presented in the supplement of Pierce et al (48). B. The green ellipse (dp/dm) is the BA07 mean direction when calculated at site level and corrected for compaction-induced inclination shallowing using a blanket factor of 0.6.

Table S1. (attached separately)

Shown is a summary of paleomagnetic poles and associated Bingham parameters, or dp/dm, from this and previous studies. Bingham ellipses calculated from past studies yield reasonable results and show the viability of using the method presented in this paper. Abbreviations: N = number of site means used to calculate pole, n = number of samples used to calculate pole, dp = uncertainty in longitude, dm = uncertainty in latitude, Bingham parameters: tmaj longitude = longitude of the ellipse major axis, tmaj latitude= latitude of ellipse major axis, amaj = ellipse major axis uncertainty, tmin longitude = longitude of ellipse minor axis, tmin latitude= latitude of ellipse minor axis, amin = ellipse minor axis uncertainty

Table S2. (attached separately)

CA-ID-TIMS results. italic = analyses omitted from the weighted mean dates. a = Corrected for initial Th/U disequilibrium using radiogenic 208Pb and Th/U[magma] specified. b = Isotopic dates calculated using λ238 = 1.55125E-10 (*81*) and λ235 = 9.8485E-10 (*81*). c = % discordance = 100 - (100 * (^{206}Pb/^{238}U date) / (^{207}Pb/^{206}Pb date)). d = Th contents calculated from radiogenic ^{208}Pb and ^{230}Th-corrected ^{206}Pb/^{238}U date of the sample, assuming concordance between U-Pb Th-Pb systems. e = Total mass of radiogenic Pb. F = Total mass of common Pb. G = Ratio of radiogenic Pb (including ^{208}Pb) to common Pb. H = Th/U ratio of magma from which mineral crystallized. i = Measured ratio corrected for fractionation and spike contribution only. j = Measured ratios corrected for fractionation, tracer and blank.

Table S3. (attached separately)

Site mean directions from stable high temperature characteristic directions, their associated statistics, and site locations. Asterisk denotes directions that are tilt corrected. Site coordinates are in degrees north and east.

Table S4. (attached separately)

Mean directions after grouping by flow unit and unique paleomagnetic direction. The site names correspond to Table S3.

Data S1. (attached separately)

A compressed folder with a self-contained GPlates project, used to generate Figure 3, is included as supplementary data to this contribution.

Data S2. (attached separately)

A text file containing the script for the age model used in the manuscript.

REFERENCES AND NOTES

1. M. Domeier, B. Robert, J. G. Meert, E. V. Kulakov, P. J. A. McCausland, R. I. F. Trindade, T. H. Torsvik, The enduring Ediacaran paleomagnetic enigma. *Earth-Sci. Rev.* **242**, 104444 (2023).

2. A. Abrajevitch, R. Van der Voo, Incompatible Ediacaran paleomagnetic directions suggest an equatorial geomagnetic dipole hypothesis. *Earth Planet. Sci. Lett.* **293**, 164–170 (2010).

3. H. C. Halls, A. Lovette, M. Hamilton, U. Söderlund, A paleomagnetic and U–Pb geochronology study of the western end of the Grenville dyke swarm: Rapid changes in paleomagnetic field direction at ca. 585 Ma related to polarity reversals? *Precambrian Res.* **257**, 137–166 (2015).

4. J. G. Meert, N. M. Levashova, M. L. Bazhenov, E. Landing, Rapid changes of magnetic field polarity in the late Ediacaran: Linking the Cambrian evolutionary radiation and increased UV-B radiation. *Gondw. Res.* **34**, 149–157 (2016).

5. B. Robert, F. Corfu, M. Domeier, O. Blein, Evidence for large disturbances of the Ediacaran geomagnetic field from West Africa. *Precambrian Res.* **394**, 107095 (2023).

6. P. J. A. McCausland, F. Hankard, R. Van der Voo, C. M. Hall, Ediacaran paleogeography of Laurentia: Paleomagnetism and ^{40}Ar–^{39}Ar geochronology of the 583Ma Baie des Moutons syenite, Quebec. *Precambrian Res.* **187**, 58–78 (2011).

7. E. I. Tanczyk, P. Lapointe, W. A. Morris, P. W. Schmidt, A paleomagnetic study of the layered mafic intrusion at Sept-Îles, Quebec. *Can. J. Earth Sci.* **24**, 1431–1438 (1987).

8. S. A. Pisarevsky, J. B. Murphy, P. A. Cawood, A. S. Collins, Late Neoproterozoic and Early Cambrian palaeogeography: Models and problems. *Geol. Soc. Lond.* **294**, 9–31 (2008).

9. N. M. Levashova, I. V. Golovanova, D. V. Rudko, K. N. Danukalov, S. V. Rudko, S. R. Yu, J. G. Meert, Late Ediacaran magnetic field hyperactivity: Quantifying the reversal frequency in the Zigan Formation, Southern Urals, Russia. *Gondwana Res.* **94**, 133–142 (2021).

10. K. P. Kodama, Combined magnetostratigraphy from three localities of the Rainstorm Member of the Johnnie Formation in California and Nevada, United States calibrated by cyclostratigraphy: A 13 R/Ma reversal frequency for the Ediacaran. *Front. Earth Sci.* **9**, 764714 (2021).

11. R. K. Bono, J. A. Tarduno, F. Nimmo, R. D. Cottrell, Young inner core inferred from Ediacaran ultra-low geomagnetic field intensity. *Nat. Geosci.* **12**, 143–147 (2019).

12. D. A. Evans, True polar wander, a supercontinental legacy. *Earth Planet. Sci. Lett.* **157**, 1–8 (1998).

13. R. K. Bono, J. A. Tarduno, A stable Ediacaran Earth recorded by single silicate crystals of the ca. 565 Ma Sept-Îles intrusion. *Geology* **43**, 131–134 (2015).

14. B. Robert, J. Besse, O. Blein, M. Greff-Lefftz, T. Baudin, F. Lopes, S. Meslouh, M. Belbadaoui, Constraints on the Ediacaran inertial interchange true polar wander hypothesis: A new paleomagnetic study in Morocco (West African Craton). *Precambrian Res.* **295**, 90–116 (2017).

15. I. Rose, B. Buffett, Scaling rates of true polar wander in convecting planets and moons. *Phys. Earth Planet. In.* **273**, 1–10 (2017).

16. G. J. Walsh, F. Benziane, J. N. Aleinikoff, R. W. Harrison, A. Yazidi, W. C. Burton, J. E. Quick, A. Saadane, Neoproterozoic tectonic evolution of the Jebel Saghro and Bou Azzer—El Graara inliers, eastern and central Anti-Atlas, Morocco. *Precambrian Res.* **216–219**, 23–62 (2012).

17. O. Blein, T. Baudin, A. Soulaimani, A. Cocherie, P. Chèvremont, H. Admou, H. Ouanaimi, A. Hafid, P. Razin, M. Bouabdelli, J. Roger, New geochemical, geochronological and structural constraints on the Ediacaran evolution of the south Sirwa, Agadir-Melloul and Iguerda inliers, Anti-Atlas, Morocco. *J. Afr. Earth Sci.* **98**, 47–71 (2014).

18. N. Youbi, R. E. Ernst, R. N. Mitchell, M. A. Boumehdi, W. El Moume, A. A. Lahna, M. K. Bensalah, U. Söderlund, M. Doblas, C. C. G. Tassinari, “Preliminary appraisal of a

correlation between glaciations and large igneous provinces over the past 720 million years" in *Geophysical Monograph Series*, R. E. Ernst, A. J. Dickson, A. Bekker, Eds. (Wiley, ed. 1, 2021), pp. 169–190.

19. M. Ousbih, M. Ikenne, B. Cousens, C. Chelle-Michou, H. El Bilali, A. Gaouzi, S. Markovic, F. Askkour, M. Mouhajir, S. El Mouden, N. Youbi, R. Ernst, Stratigraphy, geochronology, geochemistry and Nd isotopes of the Ouarzazate group, Anti-Atlas, Morocco: Evidence of a Late Neoproterozoic LIP in the northwestern part of the West African Craton. *Lithos* **474–475**, 107593 (2024).

20. E. Vernhet, N. Youbi, E. H. Chellai, M. Villeneuve, A. El Archi, The Bou-Azzer glaciation: Evidence for an Ediacaran glaciation on the West African Craton (Anti-Atlas, Morocco). *Precambrian Res.* **196–197**, 106–112 (2012).

21. N. Youbi, R. E. Ernst, U. Söderlund, M. A. Boumehdi, A. A. Lahna, C. C. G. Tassinari, W. E. Moume, M. K. Bensalah, "The Central Iapetus magmatic province: An updated review and link with the ca. 580 Ma Gaskiers glaciation" in *Mass Extinctions, Volcanism, and Impacts: New Developments* (Geological Society of America, 2020), pp. 35–66.

22. A. P. Roberts, M. Winklhofer, Why are geomagnetic excursions not always recorded in sediments? Constraints from post-depositional remanent magnetization lock-in modelling. *Earth Planet. Sci. Lett.* **227**, 345–359 (2004).

23. C. Bingham, An antipodally symmetric distribution on the sphere. *Ann. Statist.* **2**, 1201–1225 (1974).

24. T. C. Onstott, Application of the Bingham distribution function in paleomagnetic studies. *J. Geophys. Res.* **85**, 1500–1510 (1980).

25. U. Linnemann, A. P. Pidal, M. Hofmann, K. Drost, C. Quesada, A. Gerdes, L. Marko, A. Gärtner, J. Zieger, J. Ulrich, R. Krause, P. Vickers-Rich, J. Horak, A ~565 Ma old glaciation in the Ediacaran of peri-Gondwanan West Africa. *Int. J. Earth. Sci.* **107**, 885–911 (2018).

26. U. Linnemann, M. Hofmann, A. Gärtner, J. Gärtner, J. Zieger, R. Krause, R. Haenel, K. Mende, M. Ovtcharova, U. Schaltegger, P. Vickers-Rich, An Upper Ediacaran glacial period in Cadomia: The Granville tillite (Armorican Massif) – sedimentology, geochronology and provenance. *Geol. Mag.* **159**, 999–1013 (2022).

27. R. Wang, B. Shen, X. Lang, B. Wen, R. N. Mitchell, H. Ma, Z. Yin, Y. Peng, Y. Liu, C. Zhou, A Great late Ediacaran ice age. *Natl. Sci. Rev.* **10**, nwad117 (2023).

28. J. G. Meert, R. Van Der Voo, T. W. Payne, Paleomagnetism of the Catoctin volcanic province: A new Vendian-Cambrian apparent polar wander path for North America. *J. Geophys. Res.* **99**, 4625–4641 (1994).

29. A. D. Rooney, M. D. Cantine, K. D. Bergmann, I. Gómez-Pérez, B. Al Baloushi, T. H. Boag, J. F. Busch, E. A. Sperling, J. V. Strauss, Calibrating the coevolution of Ediacaran life and environment. *Proc. Natl. Acad. Sci. U.S.A.* **117**, 16824–16830 (2020).

30. G. A. Glatzmaier, R. S. Coe, L. Hongre, P. H. Roberts, The role of the Earth's mantle in controlling the frequency of geomagnetic reversals. *Nature* **401**, 885–890 (1999).

31. P. Olson, M. Landeau, E. Reynolds, True dipole wander. *Geophys. J. Int.* **215**, 1523–1529 (2018).

32. D. Thallner, A. J. Biggin, H. C. Halls, An extended period of extremely weak geomagnetic field suggested by palaeointensities from the Ediacaran Grenville dykes (SE Canada). *Earth Planet. Sci. Lett.* **568**, 117025 (2021).

33. S. Labrosse, Thermal evolution of the core with a high thermal conductivity. *Phys. Earth Planet. In.* **247**, 36–55 (2015).

34. S. Labrosse, M. Macouin, The inner core and the geodynamo. *C. R. Geosci.* **335**, 37–50 (2003).

35. W. Huang, J. A. Tarduno, T. Zhou, M. Ibañez-Mejia, L. Dal Olmo-Barbosa, E. Koester, E. G. Blackman, A. V. Smirnov, G. Ahrendt, R. D. Cottrell, K. P. Kodama, R. K. Bono, D. G. Sibeck, Y.-X. Li, F. Nimmo, S. Xiao, M. K. Watkeys, Near-collapse of the geomagnetic field

may have contributed to atmospheric oxygenation and animal radiation in the Ediacaran Period. *Commun. Earth. Environ.* **5**, 207 (2024).

36. E. V. Kulakov, C. J. Sprain, P. V. Doubrovine, A. V. Smirnov, G. A. Paterson, L. Hawkins, L. Fairchild, E. J. Piispa, A. J. Biggin, Analysis of an updated paleointensity database (Q PI -PINT) for 65–200 Ma: Implications for the long-term history of dipole moment through the Mesozoic. *J. Geophys. Res. Solid Earth* **124**, 9999–10022 (2019).

37. T. Green, S. P. Slotznick, P. Jaqueto, T. D. Raub, E. Tohver, T. E. Playton, P. W. Haines, J. L. Kirschvink, R. M. Hocking, P. Montgomery, High-resolution Late Devonian magnetostratigraphy from the Canning Basin, Western Australia: A re-evaluation. *Front. Earth Sci.* **9**, 757749 (2021).

38. A. van der Boon, A. J. Biggin, D. Thallner, M. W. Hounslow, R. Bono, J. Nawrocki, K. Wójcik, M. Paszkowski, P. Königshof, T. de Backer, P. Kabanov, S. Gouwy, R. VandenBerg, A.-C. Da Silva, A persistent non-uniformitarian paleomagnetic field in the Devonian? *Earth-Sci. Rev.* **231**, 104073 (2022).

39. D. V. Kent, B. A. Kjarsgaard, J. S. Gee, G. Muttoni, L. M. Heaman, Tracking the Late Jurassic apparent (or true) polar shift in U-Pb-dated kimberlites from cratonic North America (Superior Province of Canada). *Geochem. Geophys. Geosyst.* **16**, 983–994 (2015).

40. R. R. Fu, D. V. Kent, S. R. Hemming, P. Gutiérrez, J. R. Creveling, Testing the occurrence of Late Jurassic true polar wander using the La Negra volcanics of northern Chile. *Earth Planet. Sci. Lett.* **529**, 115835 (2020).

41. R. Van Der Voo, True polar wander during the middle Paleozoic? *Earth Planet. Sci. Lett.* **122**, 239–243 (1994).

42. R. N. Mitchell, D. A. D. Evans, T. M. Kilian, Rapid Early Cambrian rotation of Gondwana. *Geology* **38**, 755–758 (2010).

43. J. L. Kirschvink, R. E. Kopp, T. D. Raub, C. T. Baumgartner, J. W. Holt, Rapid, precise, and high-sensitivity acquisition of paleomagnetic and rock-magnetic data: Development of a

low-noise automatic sample changing system for superconducting rock magnetometers. *Geochem. Geophys. Geosyst.* **9**, 2007GC001856 (2008).

44. J. L. Kirschvink, The least-squares line and plane and the analysis of palaeomagnetic data. *Geophys. J. Int.* **62**, 699–718 (1980).

45. L. Tauxe, R. Shaar, L. Jonestrask, N. L. Swanson-Hysell, R. Minnett, A. A. P. Koppers, C. G. Constable, N. Jarboe, K. Gaastra, L. Fairchild, PmagPy: Software package for paleomagnetic data analysis and a bridge to the Magnetics Information Consortium (MagIC) Database. *Geochem. Geophys. Geosyst.* **17**, 2450–2463 (2016).

46. S. P. Lund, L. Keigwin, Measurement of the degree of smoothing in sediment paleomagnetic secular variation records: An example from late Quaternary deep-sea sediments of the Bermuda Rise, western North Atlantic Ocean. *Earth Planet. Sci. Lett.* **122**, 317–330 (1994).

47. T. R. Walker, "Diagenetic origin of continental red beds" in *The Continental Permian in Central, West, and South Europe: Proceedings of the NATO Advanced Study Institute* (Johannes Gutenberg Univ., 1976), pp. 240–282.

48. J. Pierce, Y. Zhang, E. B. Hodgin, N. L. Swanson-Hysell, Quantifying inclination shallowing and representing flattening uncertainty in sedimentary paleomagnetic poles. *Geochem. Geophys. Geosyst.* **23**, e2022GC010682 (2022).

49. R. D. Müller, J. Cannon, X. Qin, R. J. Watson, M. Gurnis, S. Williams, T. Pfaffelmoser, M. Seton, S. H. J. Russell, S. Zahirovic, GPlates: Building a virtual Earth through deep time. *Geochem. Geophys. Geosyst.* **19**, 2243–2261 (2018).

50. J. M. Mattinson, Zircon U–Pb chemical abrasion ("CA-TIMS") method: Combined annealing and multi-step partial dissolution analysis for improved precision and accuracy of zircon ages. *Chem. Geol.* **220**, 47–66 (2005).

51. D. J. Condon, B. Schoene, N. M. McLean, S. A. Bowring, R. R. Parrish, Metrology and traceability of U–Pb isotope dilution geochronology (EARTHTIME Tracer Calibration Part I). *Geochim. Cosmochim. Acta* **164**, 464–480 (2015).

52. N. M. McLean, D. J. Condon, B. Schoene, S. A. Bowring, Evaluating uncertainties in the calibration of isotopic reference materials and multi-element isotopic tracers (EARTHTIME Tracer Calibration Part II). *Geochim. Cosmochim. Acta* **164**, 481–501 (2015).

53. T. E. Krogh, A low-contamination method for hydrothermal decomposition of zircon and extraction of U and Pb for isotopic age determinations. *Geochim. Cosmochim. Acta* **37**, 485–494 (1973).

54. H. Gerstenberger, G. Haase, A highly effective emitter substance for mass spectrometric Pb isotope ratio determinations. *Chem. Geol.* **136**, 309–312 (1997).

55. J. Hiess, D. J. Condon, N. McLean, S. R. Noble, 238U/235U systematics in terrestrial uranium-bearing minerals. *Science* **335**, 1610–1614 (2012).

56. J. F. Bowring, N. M. McLean, S. A. Bowring, Engineering cyber infrastructure for U-Pb geochronology: Tripoli and U-Pb_Redux. *Geochem. Geophys. Geosyst.* **12**, DOI:10.1029/2010GC003478 (2011).

57. D. J. Condon, N. McLean, S. R. Noble, S. A. Bowring, Isotopic composition ($^{238}U/^{235}U$) of some commonly used uranium reference materials. *Geochim. Cosmochim. Acta* **74**, 7127–7143 (2010).

58. B. Schoene, J. L. Crowley, D. J. Condon, M. D. Schmitz, S. A. Bowring, Reassessing the uranium decay constants for geochronology using ID-TIMS U–Pb data. *Geochim. Cosmochim. Acta* **70**, 426–445 (2006).

59. I. Wendt, C. Carl, The statistical distribution of the mean squared weighted deviation. *Chem. Geol.* **86**, 275–285 (1991).

60. A. Von Quadt, J.-F. Wotzlaw, Y. Buret, S. J. E. Large, I. Peytcheva, A. Trinquier, High-precision zircon U/Pb geochronology by ID-TIMS using new 1013 ohm resistors. *J. Anal. At. Spectrom* **31**, 658–665 (2016).

61. J.-F. Wotzlaw, Y. Buret, S. J. E. Large, D. Szymanowski, A. Von Quadt, ID-TIMS U–Pb geochronology at the 0.1‰ level using 1013 Ω resistors and simultaneous U and18 O/16 O isotope ratio determination for accurate UO2 interference correction. *J. Anal. At. Spectrom* **32**, 579–586 (2017).

62. C.B. Keller. Chron.jl: A Bayesian framework for integrated eruption age and age-depth modelling, *OSF* (2018).

63. J. Bezanson, A. Edelman, S. Karpinski, V. B. Shah, Julia: A fresh approach to numerical computing. *SIAM Rev.* **59**, 65–98 (2017).

64. N. Etemad-Saeed, M. Hosseini-Barzi, M. H. Adabi, N. R. Miller, A. Sadeghi, A. Houshmandzadeh, D. F. Stockli, Evidence for ca. 560Ma Ediacaran glaciation in the Kahar Formation, central Alborz Mountains, northern Iran. *Gondw. Res.* **31**, 164–183 (2016).

65. B. McGee, A. S. Collins, R. I. F. Trindade, A glacially incised canyon in Brazil: Further evidence for Mid-Ediacaran glaciation? *J. Geol.* **121**, 275–287 (2013).

66. S. C. D. R. D. Souza, "Revisão estratigráfica das unidades superiores da faixa paraguai norte : Litoestratigrafia, quimioestratigrafia (C e Sr) e geocronologia (U-Pb). Mato Grosso, Brasil," thesis, Universidade de Brasília, Brasília (2015).

67. R. E. Ernst, N. Youbi, How large igneous provinces affect global climate, sometimes cause mass extinctions, and represent natural markers in the geological record. *Palaeogeogr. Palaeoclimatol. Palaeoecol.* **478**, 30–52 (2017).

68. A. Cheilletz, G. Levresse, D. Gasquet, M. Azizi-Samir, R. Zyadi, D. A. Archibald, E. Farrar, The giant Imiter silver deposit: Neoproterozoic epithermal mineralization in the Anti-Atlas, Morocco. *Miner. Deposita* **37**, 772–781 (2002).

69. D. Gasquet, G. Levresse, A. Cheilletz, M. R. Azizi-Samir, A. Mouttaqi, Contribution to a geodynamic reconstruction of the Anti-Atlas (Morocco) during Pan-African times with the emphasis on inversion tectonics and metallogenic activity at the Precambrian–Cambrian transition. *Precambrian Res.* **140**, 157–182 (2005).

70. J. D. Inglis, R. S. D'Lemos, S. D. Samson, H. Admou, Geochronological constraints on Late Precambrian intrusion, metamorphism, and tectonism in the Anti-Atlas Mountains. *J. Geol.* **113**, 439–450 (2005).

71. A. Michard, O. Saddiqi, A. Chalouan, D.F. de Lamotte. *Continental Evolution: The Geology of Morocco* (Springer, 2008) Lecture notes in earth sciences.

72. G. M. H. Ruiz, U. Helg, F. Negro, T. Adatte, M. Burkhard, Illite crystallinity patterns in the Anti-Atlas of Morocco. *Swiss J. Geosci.* **101**, 387–395 (2008).

73. A. Soulaimani, M. Burkhard, The Anti-Atlas chain (Morocco): The southern margin of the Variscan belt along the edge of the West African craton. *Spec. Publ.* **297**, 433–452 (2008).

74. T. H. Torsvik, R. Van der Voo, U. Preeden, C. Mac Niocaill, B. Steinberger, P. V. Doubrovine, D. J. J. van Hinsbergen, M. Domeier, C. Gaina, E. Tohver, J. G. Meert, P. J. A. McCausland, L. R. M. Cocks, Phanerozoic polar wander, palaeogeography and dynamics. *Earth-Sci. Rev.* **114**, 325–368 (2012).

75. G. S. Watson, A test for randomness of directions. *Geophys. J. Int.* **7**, 160–161 (1956).

76. H. Tanaka, Circular asymmetry of the paleomagnetic directions observed at low latitude volcanic sites. *Earth Planets Space* **51**, 1279–1286 (1999).

77. R. Fisher, Dispersion on a sphere. *Proc. R. Soc. Lond., Ser. A, Math. Phys. Eng. Sci.* **217**, 295–305 (1953).

78. R. Van der Voo, The reliability of paleomagnetic data. *Tectonophysics* **184**, 1–9 (1990).

79. J. G. Meert, A. F. Pivarunas, D. A. D. Evans, S. A. Pisarevsky, L. J. Pesonen, Z.-X. Li, S.-Å. Elming, S. R. Miller, S. Zhang, J. M. Salminen, The magnificent seven: A proposal for modest revision of the quality index. *Tectonophysics* **790**, 228549 (2020).

80. J. P. Pu, S. A. Bowring, J. Ramezani, P. Myrow, T. D. Raub, E. Landing, A. Mills, E. Hodgin, F. A. Macdonald, Dodging snowballs: Geochronology of the Gaskiers glaciation and the first appearance of the Ediacaran biota. *Geology* **44**, 955–958 (2016).

81. A. H. Jaffey, K. F. Flynn, L. E. Glendenin, W. C. Bentley, A. M. Essling, Precision measurement of half-lives and specific activities of ^{235}U and ^{238}U. *Phys. Rev. C* **4**, 1889–1906 (1971).
